# *LRRK2* BAC transgenic rats develop progressive, L-DOPA-responsive motor impairment, and deficits in dopamine circuit function

**DOI:** 10.1093/hmg/ddv628

**Published:** 2016-01-06

**Authors:** Max Sloan, Javier Alegre-Abarrategui, Dawid Potgieter, Anna-Kristin Kaufmann, Richard Exley, Thierry Deltheil, Sarah Threlfell, Natalie Connor-Robson, Katherine Brimblecombe, Rebecca Wallings, Milena Cioroch, David M. Bannerman, J. Paul Bolam, Peter J. Magill, Stephanie J. Cragg, Paul D. Dodson, Richard Wade-Martins

**Affiliations:** 1 Oxford Parkinson's Disease Centre,; 2 Department of Physiology, Anatomy and Genetics,; 3 Medical Research Council Brain Network Dynamics Unit, Department of Pharmacology and; 4 Department of Experimental Psychology, University of Oxford, Oxford, UK

## Abstract

Mutations in leucine-rich repeat kinase 2 (*LRRK2*) lead to late-onset, autosomal dominant Parkinson's disease, characterized by the degeneration of dopamine neurons of the substantia nigra pars compacta, a deficit in dopamine neurotransmission and the development of motor and non-motor symptoms. The most prevalent Parkinson's disease *LRRK2* mutations are located in the kinase (G2019S) and GTPase (R1441C) encoding domains of *LRRK2*. To better understand the sequence of events that lead to progressive neurophysiological deficits in vulnerable neurons and circuits in Parkinson's disease, we have generated *LRRK2* bacterial artificial chromosome transgenic rats expressing either G2019S or R1441C mutant, or wild-type LRRK2, from the complete human *LRRK2* genomic locus, including endogenous promoter and regulatory regions. Aged (18–21 months) G2019S and R1441C mutant transgenic rats exhibit L-DOPA-responsive motor dysfunction, impaired striatal dopamine release as determined by fast-scan cyclic voltammetry, and cognitive deficits. In addition, *in vivo* recordings of identified substantia nigra pars compacta dopamine neurons in R1441C *LRRK2* transgenic rats reveal an age-dependent reduction in burst firing, which likely results in further reductions to striatal dopamine release. These alterations to dopamine circuit function occur in the absence of neurodegeneration or abnormal protein accumulation within the substantia nigra pars compacta, suggesting that nigrostriatal dopamine dysfunction precedes detectable protein aggregation and cell death in the development of Parkinson's disease. In conclusion, our longitudinal deep-phenotyping provides novel insights into how the genetic burden arising from human mutant *LRRK2* manifests as early pathophysiological changes to dopamine circuit function and highlights a potential model for testing Parkinson's therapeutics.

## Introduction

Mutations in the leucine-rich repeat kinase 2 (*LRRK2/PARK8*) gene lead to the development of autosomal dominant Parkinson's disease with pathology and characteristic motor and non-motor features highly similar to sporadic forms of the disease (1,2). The *LRRK2* gene encodes a large (286 kDa), multidomain protein consisting of several repeat-containing regions, followed by ROC-COR GTPase, kinase and WD40 domains (3). Parkinson's disease-causing mutations lie in either the GTPase (R1441C/G/H), COR (Y1699C) or kinase (G2019S and I2020T) domains of the LRRK2 protein (4,5). The most common *LRRK2* mutation, G2019S, is found in about 4% of familial and 1–2% of apparently sporadic cases of Parkinson's disease (1,5); however, within certain populations, the R1441C mutation is more prevalent (6). LRRK2 has been implicated in a wide variety of cellular processes, but the mechanisms by which pathological mutations cause disease remain unclear (7).

A number of *LRRK2* transgenic mouse models have been generated to investigate the normal function of LRRK2 and its pathogenic impact in Parkinson's disease (8–20). Recently, transgenic rats expressing mutant LRRK2 have also been developed (21–23) as rats are advantageous in allowing certain electrophysiological, behavioural and imaging techniques (24). Together, these rodent models have provided important insight into the pathogenic effects of familial *LRRK2*, with most models exhibiting changes in some kind of locomotor activity and/or changes in striatal dopamine tone in the absence of progressive neurodegeneration of the substantia nigra pars compacta (SNc). However, few exhibit age-dependent motor impairment that can be linked to altered dopaminergic neuron function or altered dopamine release, consistent with a Parkinsonian phenotype.

To advance our understanding of the impact of *LRRK2* mutations on neural circuit function and the development of Parkinson's disease, we generated *LRRK2* bacterial artificial chromosome (BAC) transgenic rats, expressing either G2019S or R1441C mutant or the wild-type forms of the entire human *LRRK2* genomic locus. Our longitudinal deep-phenotyping approach allowed us to compare rats expressing mutant human LRRK2 to those with wild-type human LRRK2, and to establish whether any age-related changes in motor function were linked with progressive alterations to the nigrostriatal dopaminergic pathway. Here we report that aged *LRRK2* mutant rats develop progressive motor dysfunction which is reversed by l-DOPA, and cognitive deficits, along with impaired striatal dopamine release and alterations in the firing properties of dopaminergic SNc neurons. These impairments occur in the absence of any overt neurodegeneration or molecular neuropathology within the SNc. Overall, these findings help elucidate processes of early, pre-degenerative dopaminergic dysfunction in dopamine neurons and synapses in LRRK2-dependent Parkinson's disease.

## Results

### Molecular characterization of *LRRK2* BAC transgenic rats

**Figure 1.**
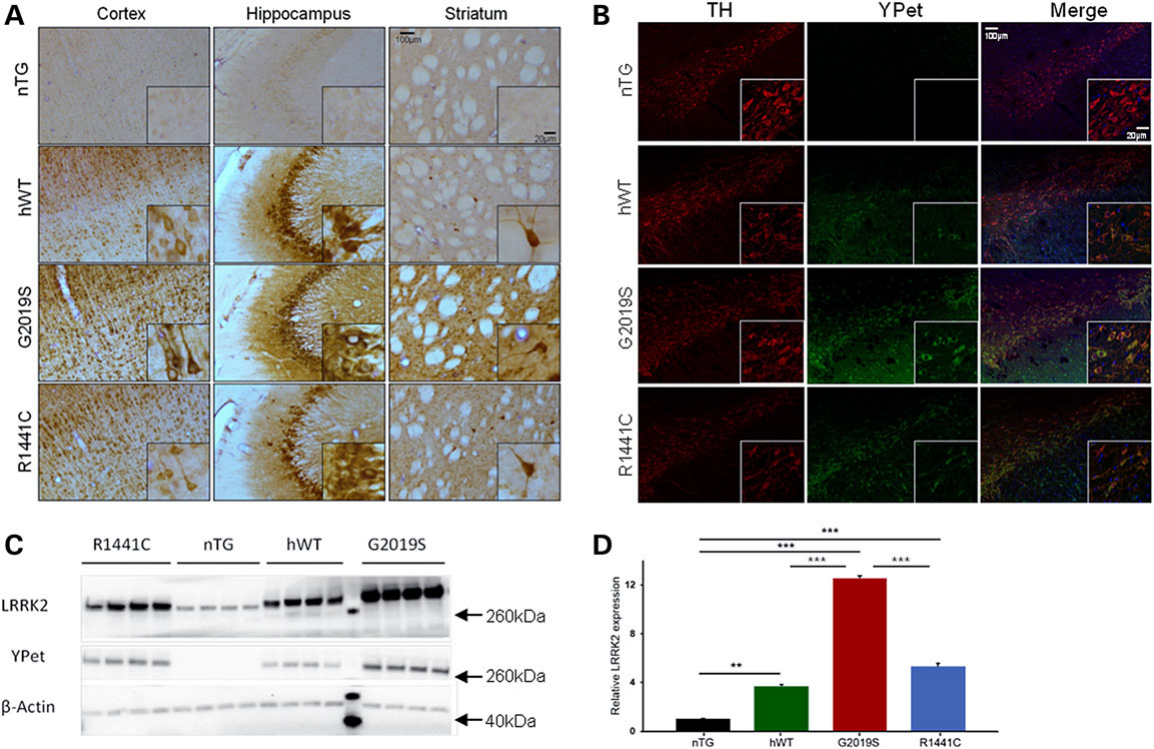
Characterization of transgene expression patterns in the brains of *LRRK2* transgenic rats. (**A**) Peroxidase-immunohistochemistry for YPet protein in cortical, hippocampal and striatal sections of 3-month-old animals from each of the transgenic lines (G2019S, R1441C, hWT) compared with an nTG control. (**B**) Double immunofluorescence labelling shows the co-localization of LRRK2-YPet fusion protein and TH in neurons of the SNc of 3-month-old animals from each of the transgenic lines (G2019S, R1441C, hWT) and a nTG control. (**C**) Whole-brain homogenate Western blots for YPet and LRRK2 in 3-month-old hWT, G2019S, R1441C and nTG rats. The molecular marker lane is run between hWT and G2019S lanes and the molecular weights of the reference bands are indicated (arrows). (**D**) Quantification of LRRK2 protein levels revealed overexpression of human LRRK2 was roughly 4–5× for hWT and R1441C, and 12× for G0291S compared with endogenous rat LRRK2 (One-way ANOVA: main effect of genotype: *P* < 0.0001; *n* = 4 per genotype). Bonferroni post hoc tests **P < 0.01, ***P < 0.001. Data are expressed as mean ± SEM.

*LRRK2* transgenic rats were generated using previously developed BAC constructs (25) consisting of the entire 144 kb human *LRRK2* locus, fused to a *YPet* reporter tag (Supplementary Material, Fig. S1A). Three *LRRK2* BAC transgenic lines were generated on a Sprague–Dawley background: one expressing human wild-type (hWT) *LRRK2* and two mutant lines, expressing either the G2019S or R1441C mutant forms of the human gene. Anatomical transgene protein expression patterns were similar between lines throughout the brain (Fig. 1A). In agreement with previous studies (26), intense staining was noted in all transgenic lines in striatal neurons morphologically identified as cholinergic interneurons. Double immunofluorescence labelling for tyrosine hydroxylase (TH) and YPet revealed transgene expression in SNc dopamine neurons at low levels for all transgenic lines (Fig. 1B). Expression levels of the hWT and R1441C LRRK2 transgenic proteins were not significantly different and at four to five times that of endogenous rat LRRK2 levels, whereas G2019S levels were 12 times that of endogenous protein levels (Fig. 1C and D). Complete transgene integration was confirmed by PCR amplification of all 51 exons using species-specific primers, and the presence of the mutations was confirmed using restriction enzyme digestion of PCR fragments (Supplementary Material, Fig. S1B–C). Fluorescent *in situ* hybridization analysis was conducted to confirm unique BAC integration sites (Supplementary Material, Fig. S1D).

Changes in phosphorylation of LRRK2 at residues ser910/935 have been suggested to mediate the interaction of LRRK2 with 14-3-3 proteins which play an important role in cellular regulatory processes (27). Mutation-dependent changes in LRRK2 phosphorylation were detected at residues ser910 and ser935, with R1441C mutants at both young and old adult time points showing greatly reduced LRRK2 phosphorylation by immunohistochemistry (Supplementary Material, Fig. S2A), subsequently confirmed by Western blot (Supplementary Material, Fig. S2B–D). Conversely, G2019S mutants showed a modest increase in phosphorylation at ser935.

### Mutant *LRRK2* rats display progressive motor and cognitive deficits

**Figure 2.**
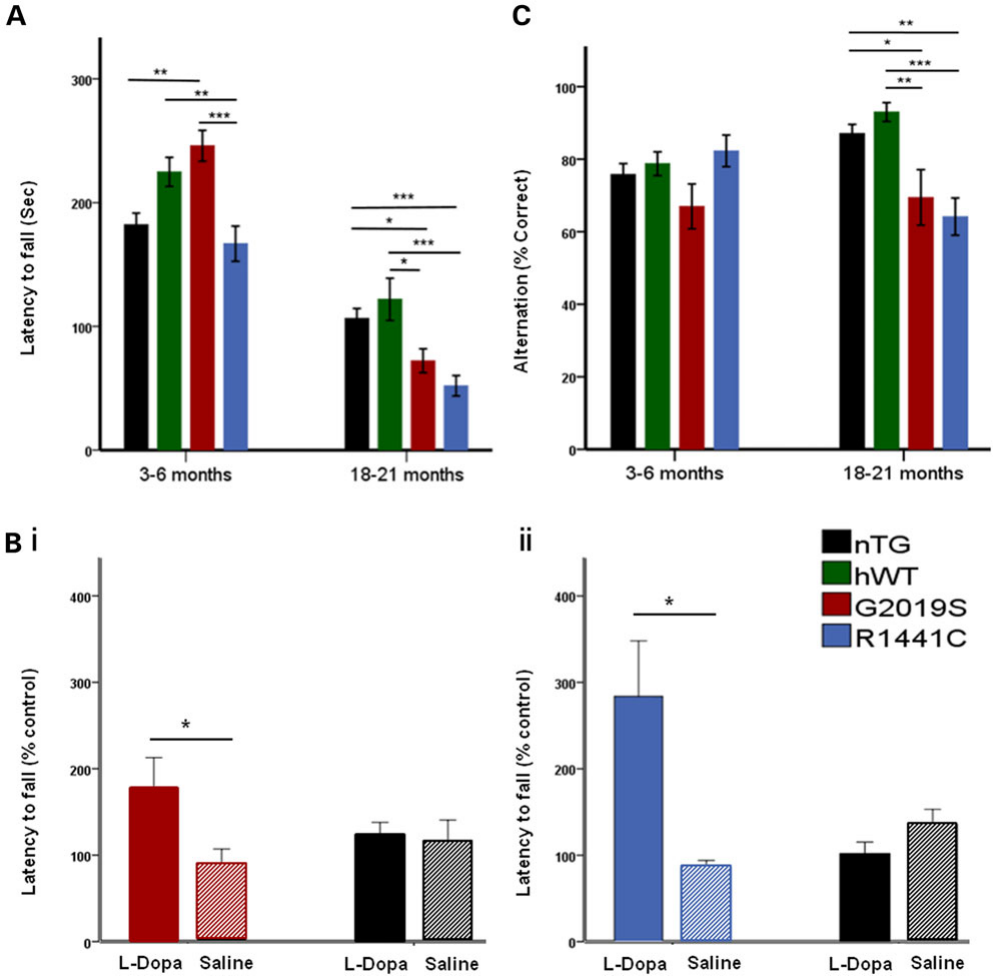
Mutant *LRRK2* transgenic rats develop late-stage l-DOPA-responsive motor deficits and cognitive impairment. (**A**) At 3–6 months G2019S, but not R1441C, animals performed better on the rotarod than nTG controls (age/genotype interaction: *P* < 0.0001). One-way ANOVA main effect of genotype, *P < 0.05, **P < 0.01, ***P < 0.001, *n* = 8 per, Tukey HSD post hoc test). At 18–21 months, rotarod performance was impaired in both G2019S and R1441C rats when compared with nTG and hWT controls (one-way ANOVA main effect of genotype, *P < 0.05, **P < 0.01, ***P < 0.001 *n* = 6–11 per genotype, Tukey HSD post hoc test. Rotarod data transformed (square-rooted to comply with parametric testing but presented as non-transformed data for ease of the reader). (**B** (**i**)) l-DOPA reversed the motor deficit in 18- to 21-month-old G2019S rats when compared with saline treatment (two-tailed Mann–Whitney *U*, *P* < 0.05, *n* = 6 per genotype). There was no significant difference in performance between l-DOPA-treated 18- to 21-month-old nTG rats and saline treated rats (two-tailed Mann–Whitney *U*, *P* > 0.05, *n* = 4 per genotype). (**B ii**) l-DOPA treatment of 21-month-old R1441C rats reversed the deficit in rotarod performance compared with saline controls (*T*-test, *P* < 0.05, *n* = 6 per genotype). Again no significant improvement in performance was seen in l-DOPA-treated nTG rats compared with saline controls (*n* = 5–6 per genotype). (**C**) Spontaneous alternation performance was impaired in old but not young *LRRK2* G2019S and R1441C mutant rats compared with nTG and hWT controls (age/genotype interaction: *P* < 0.05). At 3–6 months, performance of G2019S and R1441C animals in the spontaneous alternation test was similar compared with nTG and hWT controls (one-way ANOVA main effect of genotype, *P < 0.05, *n* = 16 per genotype, Tukey HSD post hoc test). However, at 18–21 months, *LRRK2* R1441C and G2019S rats showed significantly impaired performance on the spontaneous alternation test compared with nTG and hWT controls (one-way ANOVA main effect of genotype, *P < 0.05, **P < 0.01, ***P < 0.001, *n* = 9–14 per genotype, Tukey HSD post hoc test. Data are expressed as mean ± SEM.

Cohorts of G2019S, R1441C and non-transgenic (nTG) littermates, and of hWT and nTG littermates, were generated and underwent in-depth phenotyping. To determine whether *LRRK2* rats develop motor impairment, we analysed performance on the accelerating rotarod (Supplementary Material, Fig. S3). Compared with nTG controls at a young age (3–6 months), all transgenic lines showed no impairment on the rotarod, whereas G2019S showed an enhancement, as previously reported (23,28). At an old age (18–21 months) G2019S and R1441C mutant, but not hWT, transgenic animals showed a significant impairment in performance (Supplementary Material, Fig. S3). The non-transgenic controls in each experimental cohort showed equivalent performance allowing us to integrate data from the two cohorts to perform additional analysis directly comparing all lines (G2019S v R1441C v hWT versus nTG). Importantly, we show that aged (18–21 months) G2019S and R1441C rats exhibited a significant age-dependent impaired performance compared with both nTG and hWT controls (Fig. 2A). Gait disturbances play a major role in the motor manifestation of Parkinson's disease patients (29). We therefore examined the gait of mutant LRRK2 transgenic rats; aged R1441C, but not G2019S rats displayed abnormal gait compared with nTG controls (Supplementary Material, Fig. S4G–I).

Motor dysfunction in Parkinson's disease is clinically responsive to l-DOPA treatment owing to the loss of nigrostriatal dopamine function. To investigate whether motor impairment in the rats was l-DOPA-responsive, we administered either l-DOPA or saline to aged G2019S, R1441C and nTG rats. l-DOPA reversed the rotarod performance deficits seen in both G2019S and R1441C rats (Fig. 2B). *LRRK2* transgenic animals used for rotarod tests were not different in weight when compared with nTG controls (Supplementary Material, Fig. S4A–D). Transgenic rats did not display impaired grip strength (Supplementary Material, Fig. S4E–F), suggesting that motor deficits were likely not a consequence of muscle weakness, motor neuron alterations or motor plate neurotransmission that could potentially account for poor test performance. Taken together, these data suggest that *LRRK2* mutations result in age- and dopamine-dependent motor impairments.

The importance of non-motor symptoms of Parkinson's disease is becoming increasingly apparent. In particular, 36% of individuals with Parkinson's disease display cognitive impairment and around 40% of Parkinson's patients suffer from constipation (30,31). We therefore examined whether *LRRK2* mutant rats exhibited similar non-motor symptoms. To evaluate cognitive ability, rat performance was assessed using the spontaneous alternation test of spatial short-term memory (32). No differences in performance were seen in young adult G2019S or R1441C animals compared with controls (Fig. 2C). However, aged R1441C and G2019S rats showed significantly impaired performance on the spontaneous alternation test compared with hWT controls (Fig. 2C), which was not due to altered choice times (Supplementary Material, Fig. S5G–H). Finally, to investigate gastrointestinal function, we examined stool frequency and composition; we detected no changes in G2019S, R1441C or hWT rats at either young or aged time points compared with nTG controls (Supplementary Material, Fig. S5A–F).

### *LRRK2* BAC transgenic rats display progressive impairments in dopamine release in dorsal striatum

**Figure 3.**
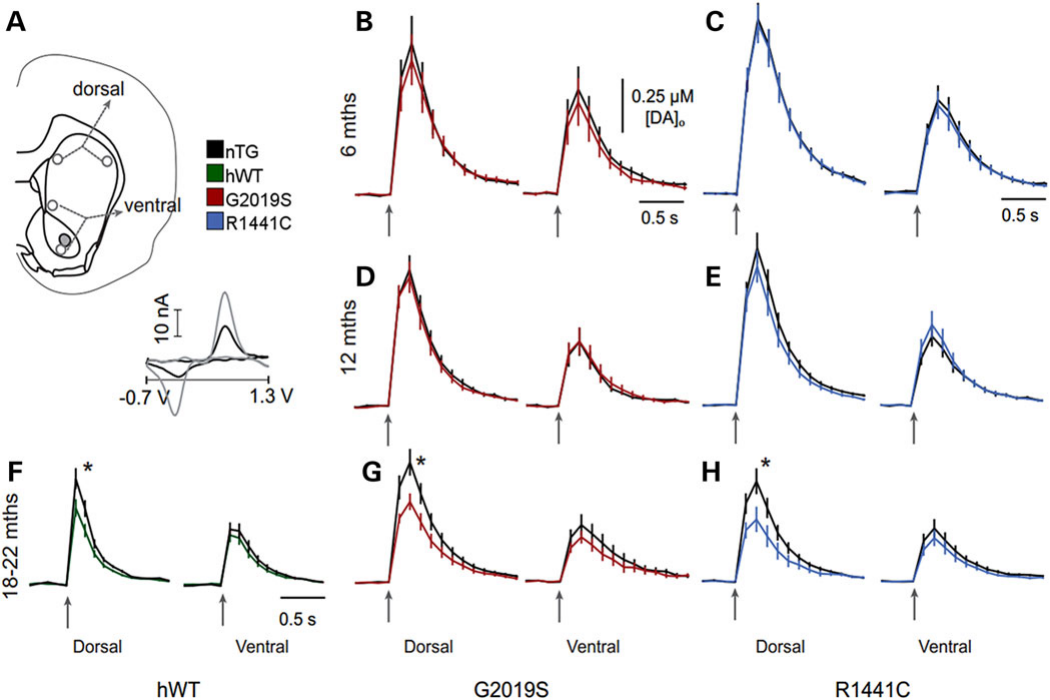
*LRRK2* transgenic rats develop late-stage dopamine transmission deficits in the dorsal striatum. (**A**) Schematic of recording site locations in striatal slices from R1441C, G2019S, hWT and nTG rats. Representative cyclic voltammogram (*inset*) recorded in striatal tissue (*black line*) shows peaks at the same oxidation and reduction potentials as a 2 µM dopamine calibration voltammogram (*grey line*). (**B–H**) Mean [DA]_o_ profiles versus time following single pulse stimulation (↑200 µs, 600 µA) in dorsal (*left panel*) or ventral striatum (*right panel*) of (**B, C**) 6-month-old G2019S and R1441C rats, (**D, E**) 12-month-old G2019S and R1441C rats and (**F–H**) 18- to 22-month-old hWT, G2019S and R1441C rats. Mean peak evoked [DA]_o_ was not significantly different between R1441C rats and nTG controls in either dorsal or ventral striatum at 6 and 12 months of age (*P* > 0.05; unpaired two-tailed Mann–Whitney; *n* = 15–29); however, at 18–21 months, mean peak evoked [DA]_o_ was significantly lower in R1441C (0.30 ± 0.06 µM) compared with nTG rats (0.49 ± 0.06 µM) in dorsal striatum (*P < 0.05; unpaired two-tailed Mann–Whitney test; *n* = 15) but not ventral striatum (*P* > 0.05; unpaired two-tailed Mann–Whitney test; *n* = 12). Mean peak evoked [DA]_o_ was not significantly different between G2019S rats and nTG controls in either dorsal or ventral striatum at 6 and 12 months of age (*P* > 0.05; unpaired two-tailed Mann–Whitney test; *n* = 11–21); however, at 18–21 months, mean peak evoked [DA]_o_ was significantly lower in G2019S rats (0.40 ± 0.04 µM) compared with nTG controls (0.59 ± 0.07 µM) in dorsal striatum (*P < 0.05; unpaired two-tailed Mann–Whitney test; *n* = 36) but not ventral striatum (*P* > 0.05; unpaired two-tailed Mann–Whitney test; *n* = 25). At 22 months, mean peak evoked [DA]_o_ was significantly lower in *LRRK2* hWT rats (0.27 ± 0.03 µM) compared with nTG controls (0.37 ± 0.04 µM) in dorsal striatum (*P < 0.05; unpaired two-tailed Mann–Whitney test; *n* = 30) but not ventral striatum (*P* > 0.05; unpaired two-tailed Mann–Whitney test; *n* = 29–30). Data are expressed as mean ± SEM. Comparison of falling phases of mean dopamine release transients from CPu of 18- to 22-month-old nTG and LRRK2 transgenic rats reveals no significant difference (one-phase exponential decay curve fits; *P* > 0.05, *n* = 15–35, *K* = 2.94–2.97 s^−1^ nTG *K* = 2.82–3.19 s^−1^ LRRK2 transgenics), suggesting no difference in reuptake rate of dopamine between genotypes.

Because Parkinson's disease patients show impairments to their striatal dopamine system (33), we considered whether dopamine transmission might be altered in mutant *LRRK2* rats. To investigate this, we used fast-scan cyclic voltammetry (FCV) at carbon-fibre microelectrodes to detect evoked dopamine release and reuptake in real time in the striatum. In Parkinson's disease, SNc neurons that innervate the dorsal striatum are preferentially affected compared with ventral tegmental area neurons innervating the ventral striatum. We therefore measured evoked dopamine release in dorsal and ventral striatum in G2019S, R1441C and hWT *LRRK2* rats compared with nTG controls. In dorsal striatum, mean peak [DA]_o_ evoked by single electrical pulses were not significantly different between either G2019S or R1441C rats and nTG controls at 6 and 12 months of age (Fig. 3A–E). However, in aged rats (18–22 months), mean peak [DA]_o_ evoked by a single pulse in dorsal striatum was 27% lower in hWT, 32% lower in G2019S, and 39% lower in R1441C rats than nTG controls (Fig. 3F–H). In contrast, in the ventral striatum, no significant difference in evoked [DA]_o_ was detected in hWT, G2019S or R1441C compared with nTG rats.

The reuptake rate of dopamine (Fig. 3F–H) and striatal dopamine content did not differ between transgenic animals and nTG controls at 6, 12 and 18–22 months (Supplementary Material, Fig. S6). Therefore, the lower levels of evoked [DA]_o_ in dorsal striatum of either G2019S, R1441C and hWT aged rats compared with nTG controls appear not to result from lower striatal dopamine content but rather to reduced ability to release dopamine. We examined whether *LRRK2* mutations reduced the probability of dopamine release by exploring the short-term plasticity of release, a measure commonly used to infer release probability owing to their typical inverse relationship at many synapse types, including for dopamine (34). We compared the ratio of [DA]_o_ evoked by four pulses at 100 Hz versus one pulse (35,36). The responses (4p:1p ratio) in dorsal striatum from aged G2019S, R1441C and hWT rats were not significantly different from nTG controls (Supplementary Material, Fig. S7), suggesting that the lower levels of evoked [DA]_o_ in dorsal striatum of G2019S, R1441C and hWT aged rats compared with nTG rats were not due to deficits in initial release probability, including release regulated by cholinergic interneuron input (36), but to other factors contributing to the ability to release dopamine, such as vesicle availability.

### Aged mutant *LRRK2* rats show no SNc dopamine neuron loss or neuropathology

**Figure 4.**
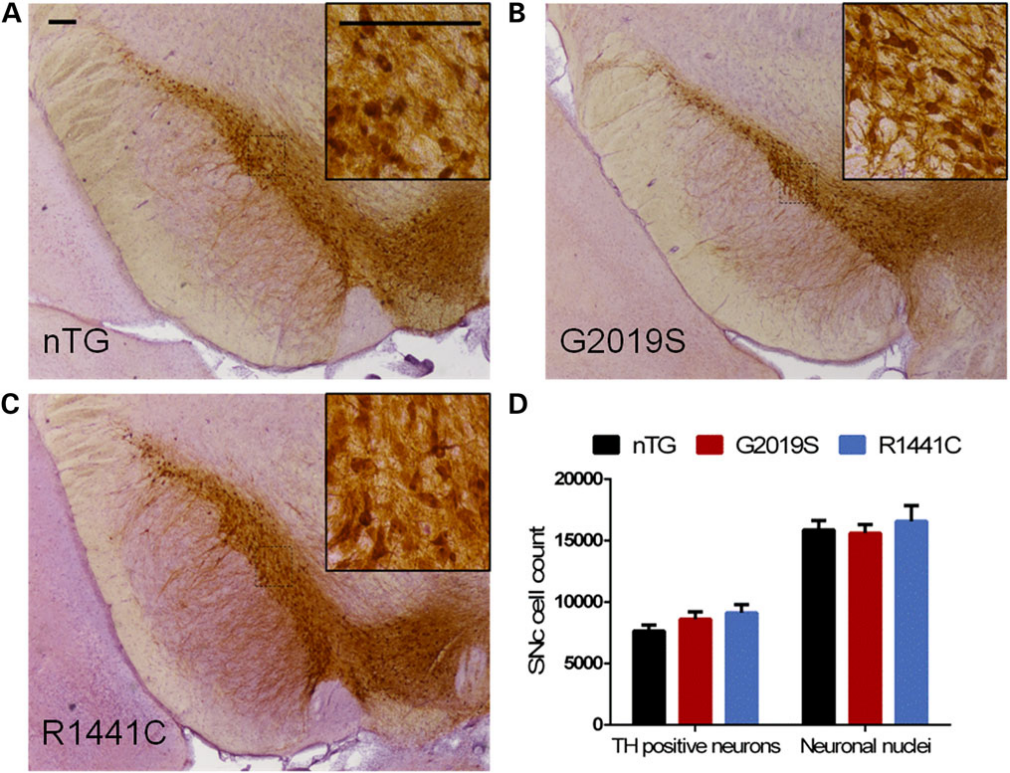
LRRK2 transgenic rats do not exhibit neurodegeneration in the SNc. (**A–C**) Sections of the LRRK2 transgenic SNc, immunolabelled for TH, that were used for stereological estimates. (**D**) Stereological estimates of the number of TH-positive neurons revealed no differences between the SNc of 18- to 21-month-old G2019S, R1441C and nTG rats. (One-way ANOVA: no main effect of genotype: *P* > 0.05, *n* = 5–10 per genotype). Similarly, there were no differences in the numbers neuronal nuclei populations between genotypes (one-way ANOVA: no main effect of genotype: *P* > 0.05, *n* = 5–10 per genotype). Scale bars, 200 µm.

To assess directly whether the behavioural changes and impaired dopamine release are a consequence of a loss of dopamine neurons in the SNc, we conducted an unbiased stereological count of TH-immunoreactive neurons in aged (18–21 month old) G2019S, R1441C and nTG control rats (Fig. 4A–C). Consistent with the observation that striatal dopamine content did not change, there was no difference in the number of TH-immunoreactive neurons, nor in the total number of SNc neurons in either mutant line compared with nTG controls (Fig. 4D). These data suggest that the observed phenotypes are not due to a loss of SNc dopamine neurons. We also investigated whether other molecules associated with striatal dopamine production and storage might be altered in aged transgenic animals. We observed no changes in expression of TH, vesicular monoamine transporter or dopamine transporter in striatum (Supplementary Material, Fig. S8). We additionally examined whether SNc neurons might express neuropathological markers prevalent in Parkinson's disease. Aged (18–21 month) animals were examined by immunohistochemistry for Parkinson's disease-related pathological molecular changes. No accumulation of immunoreactivity for alpha-synuclein, AT8, (pSer202/Thr205) tau or ubiquitin was seen in the SNc (Supplementary Material, Fig. S9). Furthermore, no changes in the number of microglial cells labelled by Iba1 staining were seen in aged *LRRK2* transgenic animals compared with nTG controls (Supplementary Material, Fig. S9).

### SNc dopamine neurons in R1441C rats show an age-dependent reduction in burst firing

**Figure 5.**
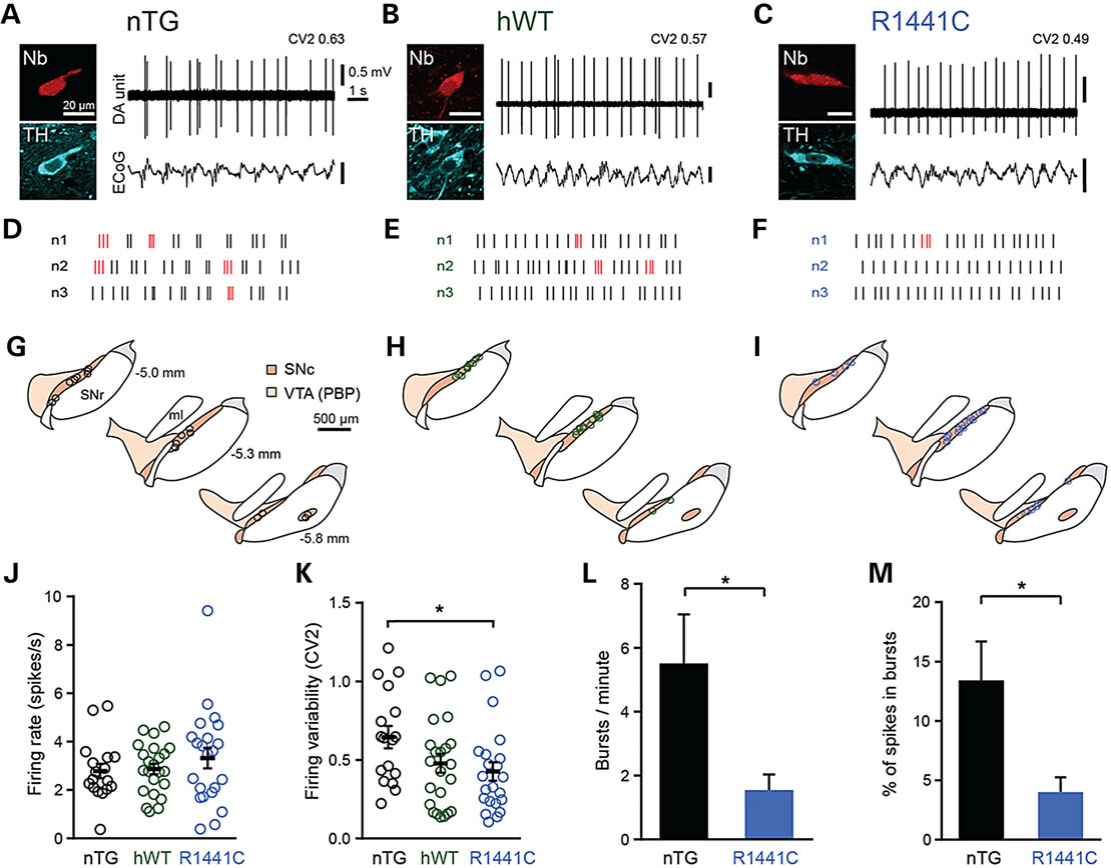
*In vivo* firing pattern of SNc dopamine neurons is more regular in aged R1441C rats. Spontaneous activity of identified SNc dopamine neurons (DA unit) in 16- to 22-month-old nTG (**A**, **D**, **G**)**,** hWT (**B**, **E**, **H**) and R1441C rats (**C**, **F**, **I**) during robust slow-wave activity (measured in the electrocorticogram, ECoG). After recording, individual neurons were juxtacellularly labelled with Neurobiotin and confirmed to be dopaminergic by expression of TH immunoreactivity. (**D**–**F**) Example raster plots denoting 10 s of spike firing in three example neurons from each genotype. Spikes detected as occurring within bursts (see Supplementary Material, methods) are highlighted in red. (**G**–**I**) Coronal schematics with approximate locations of recorded and labelled dopamine neurons for each genotype on three rostro-caudal levels (distance caudal to Bregma shown in G; dorsal top, lateral right). PBP, parabrachial pigmented area of the ventral tegmental area; SNc, substantia nigra pars compacta; SNr, substantia nigra pars reticulata; ml, medial lemniscus (adapted from (50)). (**J**,**K**) Mean firing rate and firing variability of SNc dopamine neurons (nTG *n* = 17 neurons, hWT *n* = 23 neurons, R1441C *n* = 22 neurons, *P < 0.05, one-way ANOVA on ranks, with Dunn's *post hoc* test). (**L**,**M**) Mean number of bursts per minute and mean percentage of spikes occurring within bursts (*P < 0.05, Mann–Whitney rank-sum test). Data in J–M are displayed as mean ± SEM. In (J) and (K), each circle represents data from a single neuron.

Changes in the rate or pattern of SNc dopamine neuron firing can have a profound impact on the dynamics of extracellular striatal dopamine concentrations *in vivo* (37). In theory therefore, alterations in firing could either exaggerate or even counteract the observed deficits in evoked striatal dopamine release in *LRRK2* rats (see Fig. 3) depending upon the nature of the change. We focused on R1441C rats because these exhibited the most profound deficit in striatal dopamine release. We made extracellular recordings *in vivo* from individual SNc neurons in aged (16–22 month) anaesthetized nTG and R1441C rats (Fig. 5). Following recording, we juxtacellularly labelled each neuron to verify location and dopaminergic identity (by immunoreactivity for TH; Fig. 5A–I). We first examined whether mutations in *LRRK2* influenced the firing rates of SNc dopamine neurons. We found no difference in the firing rates of SNc dopamine neurons recorded in rats of different genotypes (Fig. 5J). However, we identified a significant decrease in the variability of SNc neuron firing in R1441C rats compared with nTG controls (i.e. firing pattern becomes more regular; Fig. 5K). To determine whether changes in firing regularity were a result of the R1441C mutation in *LRRK2 per se*, or caused by elevated expression of human LRRK2, we also recorded from aged hWT rats (Fig. 5B and E). We found that the firing of SNc dopamine neurons in hWT rats was not different to that of neurons in nTG rats (Fig. 5J and K). To explore whether the decrease in firing variability in R1441C rats might be commensurate with a reduction in burst firing, we compared the occurrence of bursts in SNc neurons from R1441C rats and from nTG controls. We found that both the frequency of bursts and the percentage of spikes fired as bursts were significantly lower in R1441C rats (Fig. 5L and M). It is possible that a reduction in burst firing in aged R1441C rats might contribute to motor impairment. If this is the case, then one might not expect to observe firing regularity changes in young adult rats, because motor impairment was not evident at this age. To test this prediction, we recorded from SNc neurons in 6-month-old R1441C rats and nTG controls. There were no differences in the firing rate or regularity of SNc neurons in young rats (Supplementary Material, Fig. S10). These data suggest that the human R1441C *LRRK2* mutation significantly impacts on the firing patterns of SNc dopamine neurons in an age-dependent manner.

## Discussion

Understanding how *LRRK2* mutations impact upon the function of dopaminergic neurons is important for elucidating the key pathophysiological changes underlying the core symptoms of Parkinson's disease. Here, we describe the generation and deep-phenotyping of novel *LRRK2* BAC transgenic rat models of Parkinson's disease that express either the wild-type or R1441C or G2019S mutant forms of human LRRK2 and develop age-dependent Parkinsonian motor and non-motor symptoms. Strikingly, as seen in PD patients, motor symptoms were reversed by l-DOPA treatment, indicating that they were caused by the underlying functional alterations to the nigrostriatal dopaminergic pathway which include dysfunctional SNc dopamine neuron firing together with impaired striatal dopamine release. These alterations occur in the absence of any overt SNc neurodegeneration or pathological markers. It appears, therefore, that a reduction in dopamine neuron burst firing and dysfunctional synaptic transmission act together to reduce dopamine release and its dynamic variation in the striatum, prior to the development of molecular neuropathology or marked cell loss.

Characterization of the BAC transgenic *LRRK2* rat lines revealed widespread transgene expression that, although higher than endogenous levels in the rat, resemble expression patterns in the human brain (38,39). Between transgenic lines, anatomical expression patterns were similar, with protein expression levels between four and 12 times that of endogenous rat LRRK2, falling within the range of previously generated *LRRK2* transgenic mouse models (9,13,14,16). We additionally identified alterations in LRRK2 phosphorylation as a consequence of *LRRK2* mutation. *LRRK2* G2019S transgenic rats showed a modest increase in phosphorylation while R1441C transgenic rats showed dramatically reduced LRRK2 phosphorylation. These data are consistent with another study that found reduced LRRK2 phosphorylation in R1441C knock-in mice (27). We find that these altered phosphorylation states are not age-dependent, appearing in both young and aged rats; however, the pathological relevance of this aberrant phosphorylation remains unclear.

Parkinson's disease patients develop dysfunctional motor behaviours, including resting tremor, bradykinesia, rigidity and postural instability as well as various non-motor symptoms (40). Motor impairment (rather than general changes in locomotor activity) has only been reported in some *LRRK2* rodent models (15,22,41), and is not always shown to be age-dependent. Our behavioural characterization revealed that mutant *LRRK2* rats develop progressive motor impairment; moreover, impairment was reversed by l-DOPA, suggesting that it was caused by changes to dopaminergic circuits. We have also reported, for the first time in mutant *LRRK2* rodents, impaired cognitive function, as determined by performance on the spontaneous alternation test of short-term memory. This phenotype is interesting, given the increased incidence of cognitive impairment reported in both sporadic and *LRRK2* Parkinson's patients (30,42,43), although other studies suggest that certain forms of cognitive dysfunction may be less prevalent in *LRRK2* carriers (44,45).

Parkinson's disease patients with *LRRK2* mutations display abnormalities in presynaptic dopamine function as assessed by positron emission tomography (33). Previous use of microdialysis to investigate dopaminergic function in transgenic LRRK2 models has revealed decreased dopamine ‘tone’ in the striatum (8,16,20). Using FCV, which enables assessment of dopaminergic transmission with high spatial and temporal resolution, we have identified that mutant LRRK2 causes age-dependent impairments in dopamine release, specific to the dorsal striatum, consistent with the findings of Li *et al.* (12). Striatal dopamine release is influenced by processes affecting the dopamine axon in interaction with the rate and/or pattern of action potentials fired by dopamine neurons. For example, a burst of action potentials at higher frequency fired by an SNc neuron can cause presynaptic facilitation (35,36) and result in a greater degree of dopamine release in the striatum than by the same number of individual spikes that are not similarly clustered in time. We found that SNc neurons in aged R1441C rats fired significantly fewer bursts compared with nTG controls, which would be expected to result in attenuated dopamine release in the dorsal striatum. We predict that this effect might contribute to the more severe motor impairment observed in R1441C rats by compounding the observed deficits in the dopamine release process, resulting in even lower striatal dopamine concentrations and fewer phasic fluctuations.

Many cardinal symptoms of Parkinson's disease arise from reduced striatal dopamine (46); this is generally thought to occur as a result of the degeneration of midbrain dopaminergic neurons, especially those in the SNc. However, changes to the firing properties and dopamine release from SNc neurons in our animal models in the absence of neurodegeneration or neuropathology raise the possibility that dopamine neurons, axons and synapses in human Parkinson's disease may be dysfunctional from early stages of disease before neuronal loss. At pre-symptomatic disease stages, such circuit dysfunction might be compensated for by the many mechanisms that can govern dopamine function locally in striatum, e.g. regulation of dopamine terminals by cholinergic inputs (36,47).

The transgenic approach used to generate many rodent models relies on overexpression driven by a heterologous promotor which often results in high protein levels with non-physiological expression patterns. BAC transgenes express from the native promoter which ensures that the expression of LRRK2, although overexpressed, has the correct anatomical distribution. We tested whether elevated expression of hWT LRRK2 might be sufficient to affect the function of dopaminergic circuits. We found that expression of hWT LRRK2 resulted in impairments to dopamine release yet no motor impairment. Not only were dopamine release deficits larger in rats with *LRRK2* mutations but, in contrast to hWT rats, *LRRK2* mutant lines showed significant impairment on the rotarod and a significant reduction in dopamine neuron firing variability. Taken together, these data suggest that elevated expression of hWT LRRK2 can alter dopamine transmission but, importantly, mutations in *LRRK2* result in motor impairment and a more severe Parkinsonian phenotype.

A limitation of this study is that expression of LRRK2 in the G2019S line is around 2-fold higher than the hWT control. Consequentially, for the G2019S genotype, one cannot categorically dissociate the contribution of the increased expression and the effect of the mutant gene. However, consistently throughout our experiments, the R1441C mutation has been shown to cause a more dramatic phenotype than the G2019S mutation, despite G2019S LRRK2 being overexpressed to a greater degree. While it remains unclear whether patients with R1441C mutations develop more severe Parkinsonian symptoms than those with the G2019S mutation (1), transgenic animals with the R1441G mutation have previously shown more severe dysfunction (13,21,22).

The development of animal models which accurately recapitulate the basic pathophysiology of PD is critical for the validation of novel therapies. In this report, we have executed a broad and in-depth analysis of the effects of *LRRK2* mutation upon the mesostriatal dopamine systems using novel BAC transgenic rat models of Parkinson's disease. We have thoroughly characterized the progressive effects of *LRRK2* mutations by first identifying l-DOPA-responsive Parkinsonian behavioural phenotypes, followed by examining the effects of mutant *LRRK2* expression at multiple nodes within nigrostriatal circuits. Our deep-phenotyping approach has allowed us to determine that, prior to any overt neurodegeneration or expression of neuropathological markers, dopamine dysfunction exists at the site of dopamine release in the dorsal striatum and also in the firing properties of SNc neurons.

## Materials and Methods

### Animals

BAC transgenic rats expressing mutant or wild-type forms of the LRRK2 protein were housed with littermate controls and maintained on a 12-h light–dark cycle, with all testing conducted during the light phase. Cohort sizes were selected based on previous experience with rodent models of PD (40). All experiments and subsequent analyses were performed blind to genotype with the exception of Western blots. Old and young adult animals were tested separately and were not used across multiple time points. Groups of experimental animals from different genotypes were matched for mean age. Where a sex-dependent change was found, this is indicated in the results. If no difference was found between genders, data were pooled. For molecular analysis, including immunohistochemistry, Western blotting analysis, pathology, stereological cell counting and immunoprecipitation analysis, both male and female animals were analysed. For FCV recordings, pairs of male transgenic and nTG rats were used for recordings. Electrophysiological recordings were made from male rats. For the l-DOPA rescue experiment, animals were randomly assigned to treatment group of l-DOPA or saline. No outlier exclusion criterion was used.

### Generation of *LRRK2* BAC transgenic rats

Rat lines were created using pronuclear injection of caesium chloride-purified BAC DNA containing the *YPet*-tagged human *LRRK2* genetic locus into Sprague Dawley rat pronuclei (Unit for Laboratory Animal Medicine; University of Michigan, USA). Confirmations of full gene integration, mutation presence, germline transmission and transgene integration site were performed and three lines were selected (G2019S, R1441C and hWT). Animals were bred in a hemizygous fashion, and animals were maintained in accordance with the UK Home Office regulations under the Animals (Scientific Procedures) Act of 1986.

### Behavioural tests

#### Accelerating rotarod

Female rats were placed on the accelerating rotarod (Ugo Basile 7700) revolving at 4 rpm facing away from the experimenter. Rotor speed was incrementally increased from 4 to 40 rpm over 300 s, and the latency at which the animal fell recorded. Animals were tested three times a day for three consecutive days, and performance was averaged for each day and collapsed across days (48).

#### Effect of l-DOPA treatment on rotarod performance

Rats were administered with 10 mg/kg benzerazide, i.p. followed 30 min later with 25 mg/kg, i.p. l-DOPA. As a control, a second cohort was given equivalent volumes of saline. Animals performed rotarod testing 60 min after l-DOPA administration. Each animal's performance was normalized to the previous day's performance.

#### Spontaneous alternation within a T-maze

Animals were placed in the base of a T-maze, consisting of a start arm and two identical goal arms, surrounded by a 10 cm high wall. Each trial consisted of a sample run followed by a choice run. During the sample run, the rat was allowed a choice of one of the two goal arms. Once the animal had chosen, it was held in that goal arm for 30 s. The animal was then immediately returned to the start arm and given another free choice of either goal arm. Whether or not the animal alternated, its choice was recorded, along with the time taken for the animal to make its choice on the second run (32). Animals performed two trials per day for five consecutive days (10 trials in total).

#### Statistics

Behavioural data are expressed as means ± SEM. Analyses were performed using SPSS Statistics 20 (IBM).

### Immunohistochemistry and Western analysis

For immunohistochemistry, animals were transcardially perfused with phosphate buffer (pH 7.4) followed by 4% paraformaldehyde. Brains were subsequently cryoprotected in 30% sucrose and sliced at 35 µm (or 50 µm for stereology) using a freezing microtome. For peroxidase-immunohistochemistry, sections were incubated overnight with primary antibodies (see Supplementary Material, Table S1) followed by a biotinylated secondary antibody which was subsequently incubated in avidin–biotin–peroxidase complex and stained using diaminobenzidine. Stained sections were counterstained using haematoxylin, dehydrated and mounted. Protein extraction and Western analysis were performed as previously described in (49). Tissue was homogenized in PBS (pH 7.4) containing 1% Igepal CA-630, 0.1% SDS, 0.5% sodium deoxycholate and protease/phosphatase inhibitor mixture using a Tissue Tearor (BioSpec Products, Inc.). Protein content was quantified using a BCA assay kit (Sigma), and proteins were analysed by Western blotting. Primary antibodies used are detailed in Supplementary Material, Table S1. Bands were visualized using horseradish peroxidase-conjugated goat anti-mouse, goat anti-rabbit or goat anti-chicken IgG (Bio-Rad) and the chemiluminescent ECL+ kit (GE Healthcare). Bands were quantified using ImageJ software. Data were analysed using one-way ANOVA and Bonferroni post hoc analysis using SPSS Statistics 20 (IBM).

### Stereology

SNc neurons were counted in 50 µm sections using an unbiased, online counting, optical fractionator method using Stereo Investigator software (MicroBrightfield). Cells were counted using a Zeiss Imager M2 light microscope at 40× magnification using a randomly placed counting frame of 50 × 50 µm on a sample grid of 160 × 160 µm. A 22 µm optical dissector with ± 2 µm guard zones was used. Subregions of the SNc (lateral, dorsal, medial) were determined for counting using the Paxinos and Watson Rat Brain Atlas (6th edition; (50)). Neuronal nuclei were differentiated from other cellular nuclei based on morphology. Estimations of TH positive SNc cells and neuronal nuclei were performed in every third section from Bregma −4.56 to −6.48 mm.

### Voltammetry

Six- to 22-month-old male rats were anaesthetized with isofluorane and decapitated. Brains were removed, and coronal striatal slices (300 µm) were prepared using a vibratome (Leica VT 1200S) in ice-cold HEPES-buffered artificial cerebrospinal fluid containing: 120 mm NaCl, 5 mm KCl, 20 mm NaHCO_3_, 6.7 mm HEPES acid, 3.3 mm HEPES salt, 2 mm CaCl_2_, 2 mm MgSO_4_, 1.2 mm KH_2_PO_4_ and 10 mm glucose and saturated with 95% O_2_/5% CO_2_. After a minimum of 1 h recovery at room temperature, slices were then transferred to the recording chamber and maintained in an oxygenated bicarbonate-buffered artificial cerebrospinal fluid containing: 124 mm NaCl, 2.1 mm KCl, 26 mm NaHCO3, 2.4 mm CaCl2, 1.3 mm MgSO4, 1.3 mm KH2PO4 and 10 mm glucose as described previously (49,51–53). [DA]_o_ was monitored using FCV with 7 µm diameter carbon fibre microelectrodes (tip length 50–100 µm, fabricated in-house) and a Millar Voltammeter (Julian Millar, Barts and the London School of Medicine and Dentistry). The scanning voltage was a triangular waveform (−0.7 to +1.3 V range versus Ag/AgCl) at a scan rate of 800 V/s and sampling frequency of 8 Hz. Electrodes were positioned in brain slices to a depth of 100 μm. Amine release was evoked locally by a surface, concentric bipolar Pt/Ir electrode (25 μm diameter; FHC) placed ∼150 μm away from the carbon-fibre microelectrode. Stimulus pulses were generated out-of-phase with FCV scans and were applied at the lowest current that generated maximal dopamine release with a single stimulus pulse in wild-type animals (600 µA, 200 µs pulse duration). [DA]_o_ was monitored and quantified in dorsal and ventral striatum. The evoked current signal was confirmed as dopamine by comparing the peak potentials for oxidation and reduction currents with those of dopamine in calibration media (+500–600 and 200 mV versus Ag/AgCl, respectively). Electrodes were calibrated in 1–2 µM dopamine following experiments in all experimental media. FCV experiments assessed [DA]_o_ evoked by discrete stimuli in the dorsal striatum (dorsal) and ventral striatum (ventral). Recording sites classed as dorsal were from dorsomedial and dorsolateral striatum, whereas those classed as ventral were from ventromedial striatum and nucleus accumbens core (see Fig. 3A). Data were collected as follows: multiple sites were sampled per slice (dorsolateral, dorsomedial and ventromedial striatum and nucleus accumbens core) in both genotypes on the same experimental day. Stimuli in these experiments consisted of either a single pulse or four pulses at 25 or 100 Hz separated by 2.5 min, and alternating between genotypes (either G2019S, R1441C or hWT versus nTG). The order in which sites were recorded from was randomized between experiments. The two genotypes being compared on a given recording day would be age-matched, and the experimenter was blinded to genotype.

### *In vivo* electrophysiological recording and juxtacellular labelling of dopaminergic neurons

Experimental procedures were performed on male 6-months-old rats (six nTG and five R1441C rats) and male 16- to 22-months-old rats (six nTG, five hWT and eight R1441C rats) in accordance with the Animals (Scientific Procedures) Act of 1986 (United Kingdom). All experiments and subsequent analyses were performed blind to genotype. The sample size, *n*, is the number of observations. Anaesthesia was induced with 4% isofluorane (Isoflo; Shering-Plough), and maintained with urethane (1.2 g/kg, i.p.; ethyl carbamate, Sigma-Aldrich), with ketamine (30 mg/kg, i.p.; Ketaset, Willows Francis) and xylazine (3 mg/kg, i.p.; Rompun, Bayer) supplements given as required. Anaesthesia was monitored during experiments using the electrocorticogram (see below) and by testing reflexes to a cutaneous pinch or gentle corneal stimulation. Wound margins were infiltrated with the local anaesthetic bubivacaine (0.75% w/v, Marcaine, AstraZeneca) and corneal dehydration was prevented with application of Hypromellose eye drops (Norton Pharmaceuticals) and eye ointment (Lacri-Lube, Allergan). Protocols for electrophysiology were similar to those previously described (54): Animals were placed in a stereotaxic frame (Kopf) and their body temperature maintained at 37 ± 0.5°C using a homeothermic heating device (Harvard Apparatus). For electrocorticogram recordings, a stainless steel screw (1.4 mm diameter) was juxtaposed on the dura mater above the right frontal cortex (anterioposterior 4.2 mm and mediolateral 2 mm distance from Bregma (50) and was referenced against a screw positioned above the cerebellum. The raw electrocorticogram was bandpass-filtered (0.3–1500 Hz, −3 dB limits) and amplified (2000×, DPA-2FS filter/amplifier; NPI) prior to acquisition. A craniotomy was performed above the right and/or left SNc and the dura mater removed. Saline solution (0.9% w/v NaCl) was frequently applied around the craniotomy to prevent dehydration of the exposed cortex. Extracellular recordings of single-unit activity were made with glass electrodes (tip diameter ∼1.5 μm, *in situ* resistance 10–25 MΩ) filled with saline solution (0.5 M NaCl) and Neurobiotin (Nb) (1.5% w/v, Vector Laboratories). Electrode signals were amplified (10×) through the active bridge circuitry of an Axoprobe-1A amplifier (Molecular Devices Corp). Single-units were recorded after alternating current coupling, amplification (100×; DPA-2FS; NPI), and standard bandpass filtering (between 300 and 5000 Hz; DPA-2FS; NPI). A Humbug (Quest Scientific) was used to eliminate mains noise at 50 Hz. Spikes were several millivolts in amplitude and showed an initial positive deflection. To avoid possible sampling bias, generous online criteria were applied to guide recordings (spike duration threshold-to-trough for bandpass-filtered spikes of >0.8 ms and firing rates < 20 Hz) (49).

Following recording, single neurons were labelled with Nb using the juxtacellular method (54,55) to allow for their unambiguous identification and localization. In brief, the electrode was advanced slowly towards the neuron while a microiontophoretic current was applied (1–10 nA positive current, 200 ms duration, 50% duty cycle). Neurobiotin was then left to transport along neuronal processes for up to 12 h. At the end of the experiment, animals were given a lethal dose of anaesthetic and perfused via the ascending aorta with 200 ml of 0.01 M phosphate-buffered saline (PBS) at pH 7.4 followed by 300 ml of 4% w/v paraformaldehyde in 0.1 M phosphate buffer, pH 7.4 (PFA). Brains were left in PFA at 4°C until they were sectioned 12–72 h later.

Tissue processing was similar to that previously used to identify recorded and labelled cells in mice (49): fixed brains were sectioned at 50 μm in the coronal plane on a vibrating blade microtome (VT1000S; Leica Microsystems), collected and washed in PBS. Free-floating sections were incubated for 4 h at room temperature in PBS with 0.3% (vol/vol) Triton X-100 (PBS-Triton, Sigma-Aldrich) and Cy3-conjugated streptavidin (diluted 1:1000, PA43001, GE Healthcare). Tissue sections were then mounted on slides for viewing with an epifluorescence microscope (Axiophot, Zeiss) and sections containing Nb-labelled neuronal somata (i.e. those labelled with Cy3) were identified and isolated. All Nb-labelled neurons were tested for expression of TH. The sections were then incubated overnight at room temperature in PBS-Triton with mouse anti-TH (1:1000, T2928/T1299, Sigma-Aldrich), washed in PBS and then incubated for 4 h at room temperature in PBS-Triton with AMCA-conjugated secondary antibody to visualize immunoreactivity of TH (donkey anti-mouse IgG, 1:500; 715-155-150, Jackson Immunoresearch Laboratories). Images were acquired using a confocal laser-scanning microscope (LSM710, Zeiss) and ZEN software (2008). An appropriate set of laser beams for AMCA (excitation 405, emission 409–559) and CY3 (excitation 543, emission 538–681) were used (40×, 1.3 numerical aperture oil immersion objective). Digital images were cropped to regions of interest and brightness and contrast adjusted when needed (Photoshop software, Adobe Systems). Only neurochemically identified (TH expressing) neurons located within the SNc were included in the analysis.

All biopotentials were digitized online using a Power 1401 analogue-digital converter (Cambridge Electronic Design) and acquired using Spike2 software (version 7.12; Cambridge Electronic Design). Because the firing pattern of dopaminergic neurons within the SNc can vary with the brain state (54), only unit activity recorded during robust cortical slow-wave activity (SWA) was included in the analysis. For the extraction of periods of SWA, electrocorticogram data were Fourier-transformed (frequency resolution 0.2 Hz) and the power ratio in the SWA band (0.5–2 Hz) to the power in the gamma band (30–80 Hz) was calculated. Epochs of contiguous data with a power of >13 for each data point were concatenated for further analysis (mean duration of concatenated epochs 199.4 ± 8.1 s, *n* = 82). Putative single-unit activity was isolated using template matching, principal component analysis and supervised clustering within Spike2. Firing regularity was calculated using CV2 (56) which compares the variability of adjacent inter-spike intervals within a spike train, then a mean CV2 value was obtained for each neuron (lower CV2 values indicate more regular unit activity). Bursts in dopaminergic SNc neurons were determined using the robust Gaussian surprise method (RGS) (57), a statistical method to detect clusters of spikes with significantly lower ISI's than a central distribution obtained from the pooled and normalized spike trains. Percentage of total numbers of spikes occurring in bursts were calculated from RGS obtained clusters. RGS parameters were set to a minimum of three spikes per burst. The Shapiro–Wilk test was used to judge whether datasets were normally distributed (*P* < 0.05 to reject). If data failed normality tests, a Mann–Whitney *U*-test was used (SigmaStat, Systat Software Inc.). Significance for all statistical tests was set at *P* < 0.05.

### Statistical analysis

Data are expressed as mean ± SEM. Appropriate tests are used (Welch's *t*-test, one/two-way ANOVA with Bonferroni or Tukey HSD post hoc tests, *t*-test, Mann–Whitney). Where appropriate, data were transformed (square-rooted) so as to comply with parametric testing, where this was done the data has been presented as non-transformed for the ease of the reader. Separate cohorts of G2019S, R1441C and non-transgenic littermates, and of hWT and non-transgenic littermates, were generated and underwent in-depth phenotyping. Where transgenic animals showed a significant difference from non-transgenic littermate controls in performance on the rotarod and in the spontaneous alternation tests the data were combined, and the performance of the three transgenic lines (G2019S v R1441C v hWT) could be directly compared, as the respective nTG control groups did not differ statistically from each other. For completeness, the original non-combined data are represented in Supplementary Material, Figure S3.

## Supplementary Material

Supplementary Material is available at *HMG* online.

## Funding

The work was supported by the Monument Trust Discovery Award from Parkinson's UK (grant no. J-0901), and the Medical Research Council (MRC) UK (awards nos. U138197109, MC_UU_12020/5 and MC_UU_12024/2 to P.J.M., and awards U138164490 and MC_UU_12020/1 to J.P.B.). M.S. and R.W. hold a Joan Pitts-Tucker/Moritz studentship; A.K. and D.P. hold MRC studentships and D.M.B. held a Wellcome Trust Senior Research Fellowship. Funding to pay the Open Access publication charges for this article was provided by the Charity Open Access Fund.

## Authors’ Contributions

M.S., J.A.-A., D.P., A.K., S.T., N.C.-R., J.P.B., P.J.M., S.J.C., P.D.D., and R.W.-M. designed research; M.S., J.A.-A., D.P., A.K., R.E., S.T., T.D., N.C.-R., K.B., R.W., M.C. and P.D.D. performed research; M.S., D.P., A.K., S.T., N.C.-R., R.W., D.M.B. and P.D.D. analysed data; M.S., S.T., N.C.-R., P.D.D. and R.W.-M. wrote the paper.

## Supplementary Material

Supplementary Data
