# Supplementary material for: LRRK2 BAC transgenic rats develop progressive, L-DOPA-responsive motor impairment, and deficits in dopamine circuit function
Source: Hum Mol Genet. 2016 Jan 6;25(5):951–63. doi: 10.1093/hmg/ddv628 (PMC4754049; doi:10.1093/hmg/ddv628)
Supplement: Supplementary Data [file supp_25_5_951__index.html]

LRRK2 BAC transgenic rats develop progressive, L-DOPA-responsive motor impairment, and deficits in dopamine circuit function — LRRK2 BAC transgenic rats develop progressive, L-DOPA-responsive motor impairment, and deficits in dopamine circuit function — Supplementary Data 

# *LRRK2* BAC transgenic rats develop progressive, L-DOPA-responsive motor impairment, and deficits in dopamine circuit function

## Supplementary Data

Supplementary Data

- Supplementary Data - Pdf file
