# Supplementary material for: LRRK2 BAC transgenic rats develop progressive, L-DOPA-responsive motor impairment, and deficits in dopamine circuit function
Source: Hum Mol Genet. 2016 Jan 6;25(5):951–63. doi: 10.1093/hmg/ddv628 (PMC4754049; doi:10.1093/hmg/ddv628)
Supplement: Supplementary Data [file supp_ddv628_ddv628supp.pdf]

SUPPLEMENTARY FIGURES

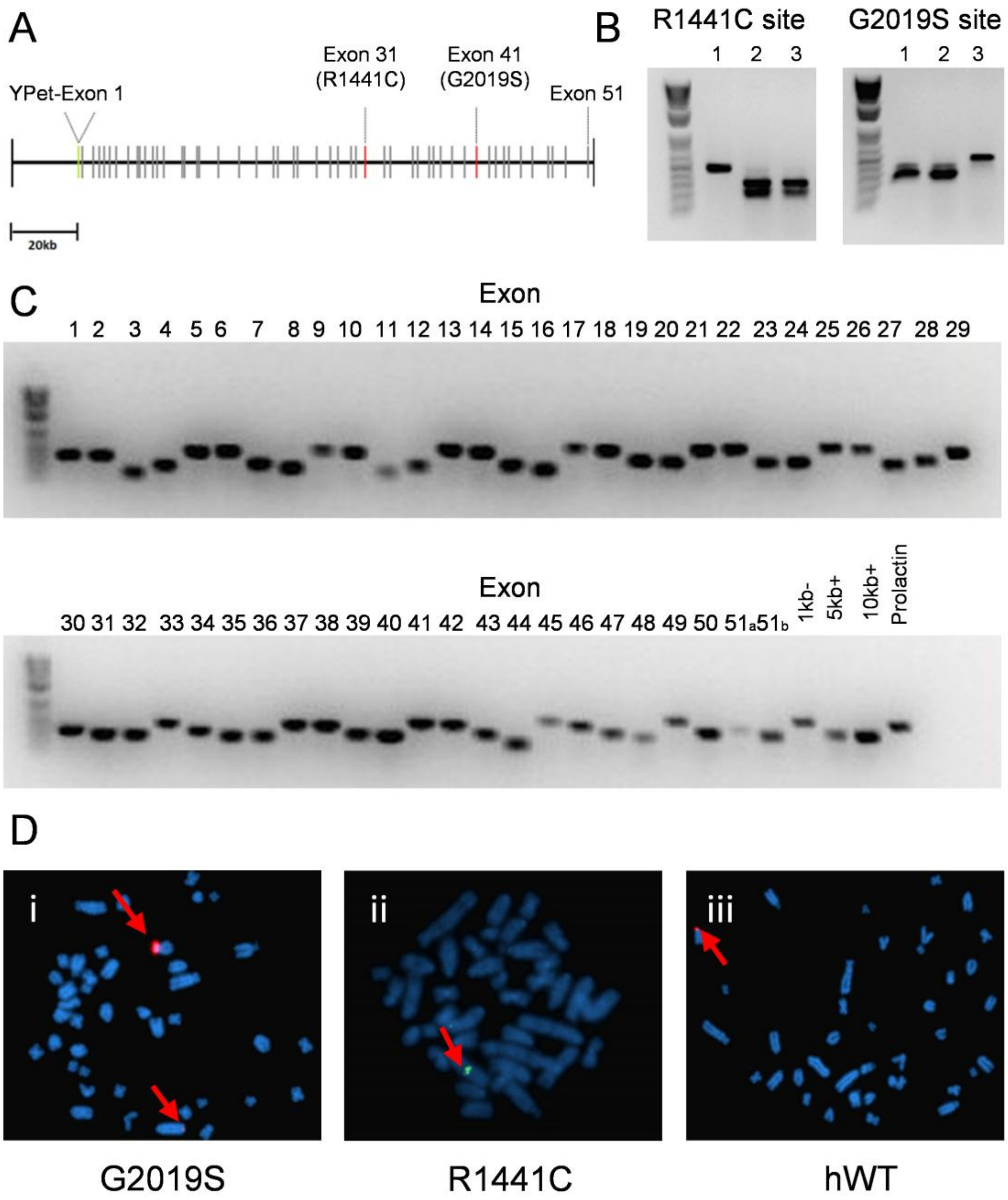

Figure S1. Verification of entire LRRK2 gene, mutations and transgene integration sites. (**A**) Linear representation of the LRRK2-YPet BAC containing the complete human *LRRK2* genomic locus (144 kb) with an N-terminally tagged *YPet* fused to exon 1. Upstream sequences are ~20 kb and downstream sequences are ~1kb in length. (**B**) Example of a restriction

digest assessing the presence of mutations at exon 31 (R1441C) and exon 41 (G2019S) of the LRRK2 gene. Presence of the R1441C mutation eliminates a BstU1 site while presence of a G2019S mutation eliminates a BceA1 site. DNA cleavage therefore indicates a wild-type genotype. (**C**) Example of high resolution PCR of a rat founder, verifying the presence of the complete *LRRK2-YPet* transgene. Lane numbers correspond to respective exons. Two primer pairs were designed for exon 51. Primer pairs targeting 5 kb and 10 kb upstream and 1 kb downstream were also used. (**D**) Fluorescent in situ hybridization analysis showing integration sites in fibroblasts in the (**i**) G2019S line, showing a centromeric integration in an acrocentric chromosome plus a small signal on the subtelemeric region of a larger acrocentric chromosome, (**ii**) R1441C line, showing integration in an acrocentric chromosome and (**iii**) hWT line, showing a centromeric integration site in an acrocentric chromosome.

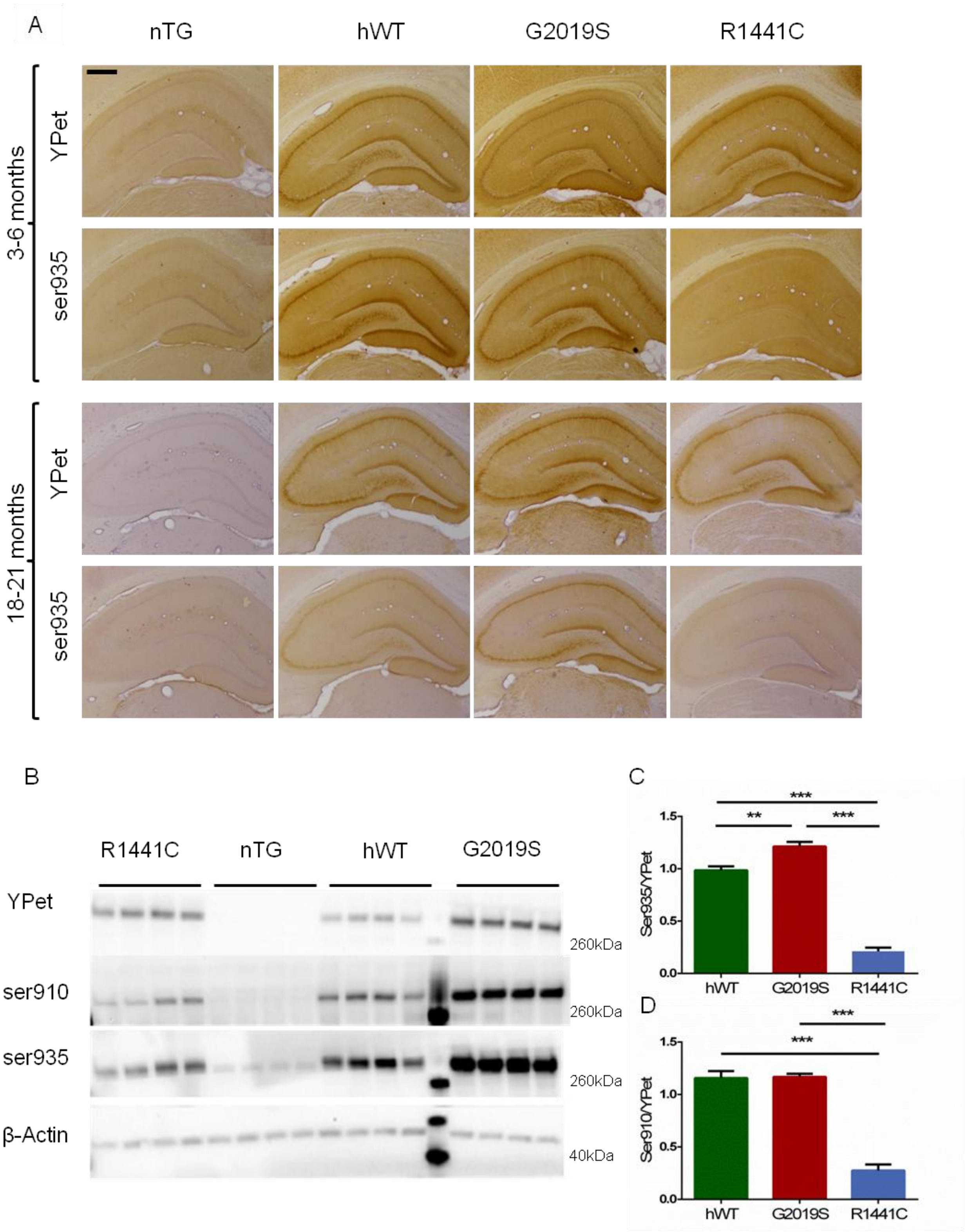

Figure S2. G2019S and R1441C rats show altered levels of LRRK2 phosphorylation.

(**A**) Immunohistochemistry at both young and old adult ages shows that R1441C rats have

reduced ser935 LRRK2 phosphorylation in hippocampus. Scale bar, 500 µm. (**B-D**) R1441C rats show reduced ser910 (One-way ANOVA: main effect of genotype: $p < 0.0001$; n=4 per genotype) and ser935 (One-way ANOVA: main effect of genotype: $p < 0.0001$; n=4 per genotype) LRRK2 phosphorylation and G2019S rats show increased ser935 LRRK2 phosphorylation via Western blot. Data are expressed as mean ± SEM. Bonferroni post-hoc tests ** $p < 0.01$, ***$p < 0.001$.

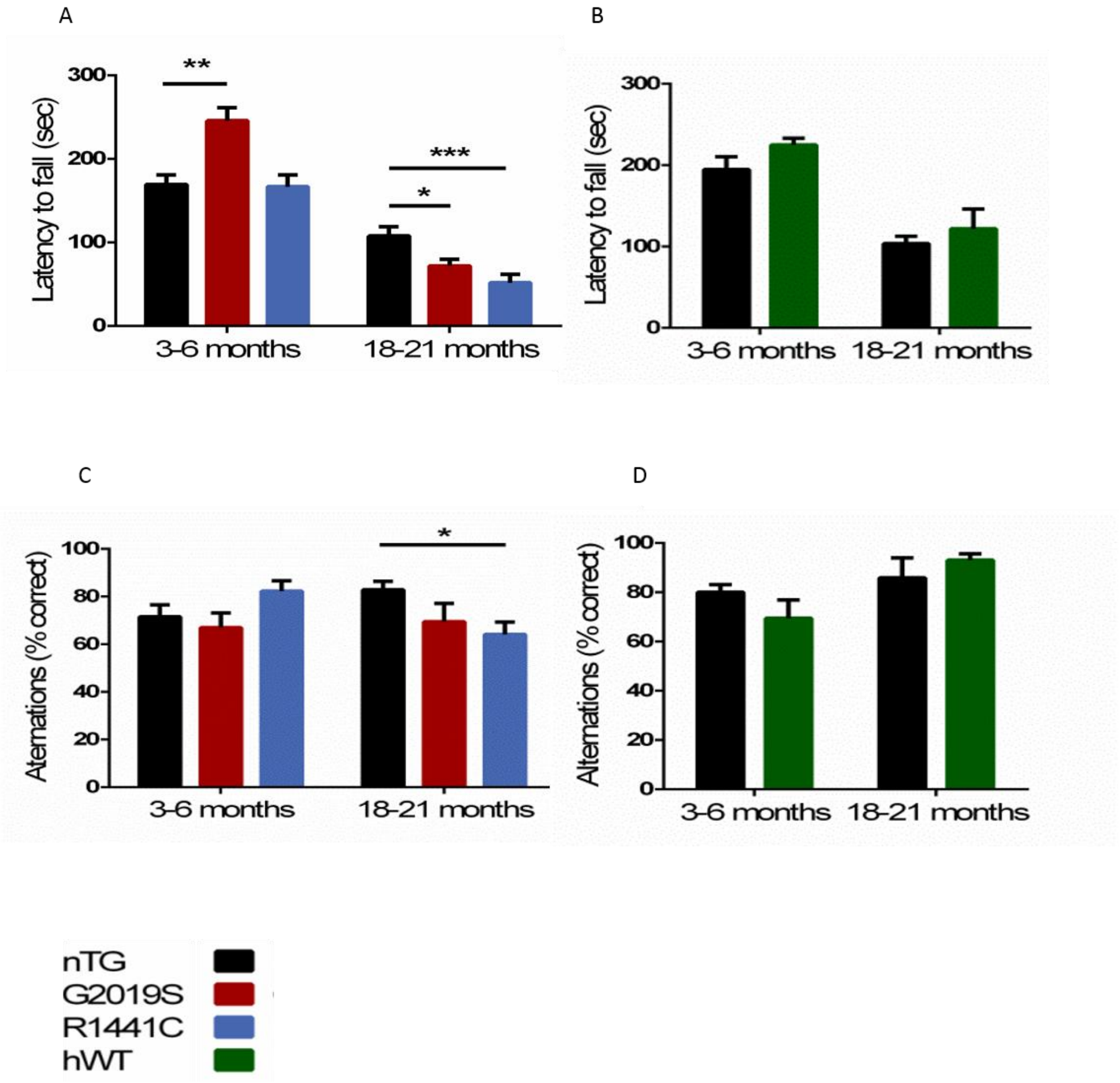

Figure S3 Aged LRRK2 mutant rats demonstrate rotarod and spontaneous alternation deficits. Original data from cohorts of G2019S, R1441C and non-transgenic littermates, and of hWT and non-transgenic littermates, shown in combined form in Fig 2. (**A**) Rotarod performance was impaired in 18-21 but not 3-6 month old mutant LRRK2 rats (Age/genotype interaction: $p < 0.0001$). At 3-6 months, G2019S but not R1441C animals performed consistently better on the

rotarod than nTG controls (One-way ANOVA, $p < 0.01$, n=8 per genotype). At 18-21 months, rotarod performance was impaired in both G2019S and R1441C rats when compared to nTG controls (One-way ANOVA, $p < 0.01$; n = 8-11 per genotype. (**B**) No difference in rotarod performance was seen in 3-6 month old hWT animals compared to nTG controls (t-test, $p > 0.05$, n=8 per genotype). Similarly, no difference in rotarod performance was seen in 18-21 month old hWT animals compared to nTG controls (t-test, $p > 0.05$, n=6-7 per genotype). (**C**) Spontaneous alternation performance was impaired in 18-21 month old R1441C mutants but not 3-6 month old R1441C or G2019S rats, compared to nTG controls (Age/genotype interaction: $p < 0.05$). At 3-4 months, performance of G2019S and R1441C animals in the spontaneous alternation test was similar compared to nTG controls (One-way ANOVA, $p > 0.05$, n=16 per genotype). At 18-21 months, R1441C but not G2019S rats showed significantly impaired performance on the spontaneous alternation test compared to nTG controls (One-way ANOVA, $p < 0.05$, n=9-14 per genotype). (H) No difference in performance in the spontaneous alternation test was seen in 3-6 (t-test, $p > 0.05$, n=16 per genotype) or 18-21 month old (t-test, $p > 0.05$, n=9-12 per genotype) hWT animals compared to nTG controls. Dunnett posthoc tests * $p < 0.05$, ** $p < 0.01$, ***$p < 0.001$. Data are expressed as mean ± SEM.

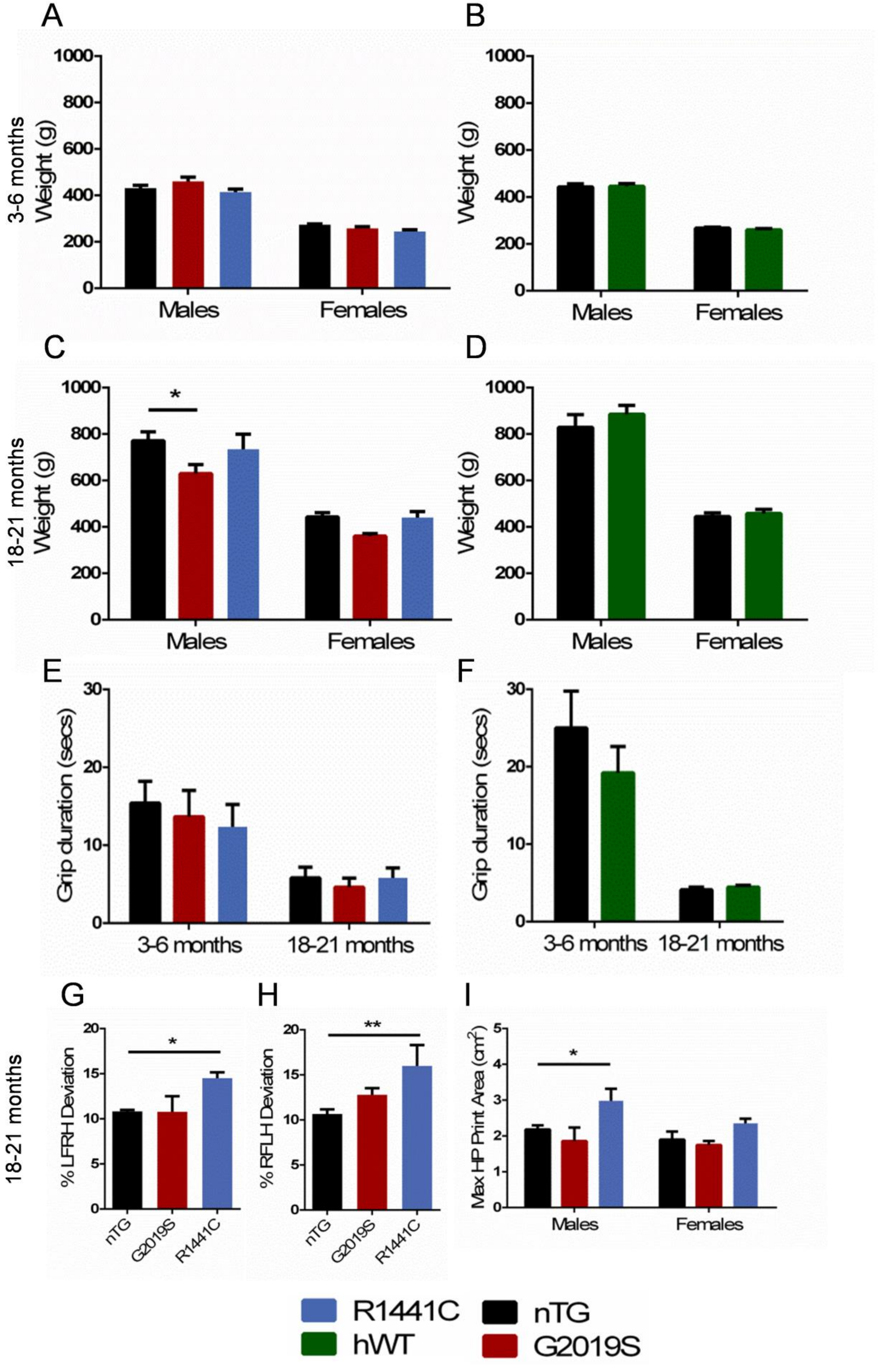
A
B
C
D
E
F
G
H
I
3-6 months
18-21 months
18-21 months
Weight (g)
Males
Females
Grip duration (secs)
3-6 months
18-21 months
% LFRH Deviation
% RFLH Deviation
Max HP Print Area (cm2)
nTG
G2019S
R1441C
hWT
*
**

Figure S4. Mutant *LRRK2* rat weights, grip strength and gait analyses. (**A**) Young G2019S and R1441C male and female animals showed no significant difference in weight compared to age-matched nTG rats (two-way ANOVA: main effect of gender: $p < 0.0001$; no main effect of genotype: $p > 0.05$, n=16 per genotype). (**C**) Aged male G2019S but not R1441C animals showed decreased weight compared to age-matched nTG rats (two-way ANOVA: main effect of gender: $p < 0.0001$; main effect of genotype: $p < 0.001$, n=9-14 per genotype). Neither young hWT (**B**) (two-way ANOVA: main effect of gender: $p < 0.0001$; no main effect of genotype: $p > 0.05$; n=16 per genotype) nor aged hWT (**D**) (two-way ANOVA: main effect of gender: $p < 0.0001$; no main effect of genotype: $p>0.05$; n=9-12 per genotype) animals show significant differences in weight compared to age-matched nTG rats. (**E**) Young G2019S and R1441C animals showed no change in latency to fall compared to age-matched nTG rats in the grip strength test (One-way ANOVA: no main effect of genotype: $p > 0.05$; n=8 per genotype). Similarly, aged G2019S and R1441C animals showed no change in latency to fall compared to age-matched nTG rats (One-way ANOVA: no main effect of genotype: $p > 0.05$; n=8-11 per genotype). (**F**) Neither young hWT (Welch's t-test: $p > 0.05$; n=8 per genotype) nor aged hWT (Welch's t-test: $p > 0.05$; n=5-6 per genotype) animals showed any change in latency to fall compared to age-matched nTG rats**.** (**G-H**) Aged R1441C but not G2019S animals showed increased (**G**) LFRH (One-way ANOVA: main effect of genotype: $p < 0.05$; n=9-14 per genotype) and (**H**) RFLH (One-way ANOVA: main effect of genotype: $p < 0.01$; n=9-14 per genotype) phase dispersion compared to age-matched nTG rats. (**I**) Aged R1441C but not G2019S animals show increased max HP print area (two-way ANOVA: no main effect of gender: $p > 0.05$; main effect of genotype: $p < 0.01$, n=9-14 per genotype) compared to age-matched nTG rats. Data are expressed as mean ± SEM. Dunnett post-hoc tests * $p < 0.05$, ** $p < 0.01$.

HP= hind paw, LFRH=left forepaw right hind paw, RFLH=right forepaw left hind paw. Data are expressed as mean ± SEM. Dunnett post-hoc tests * $p < 0.05$ ** $p < 0.01$.

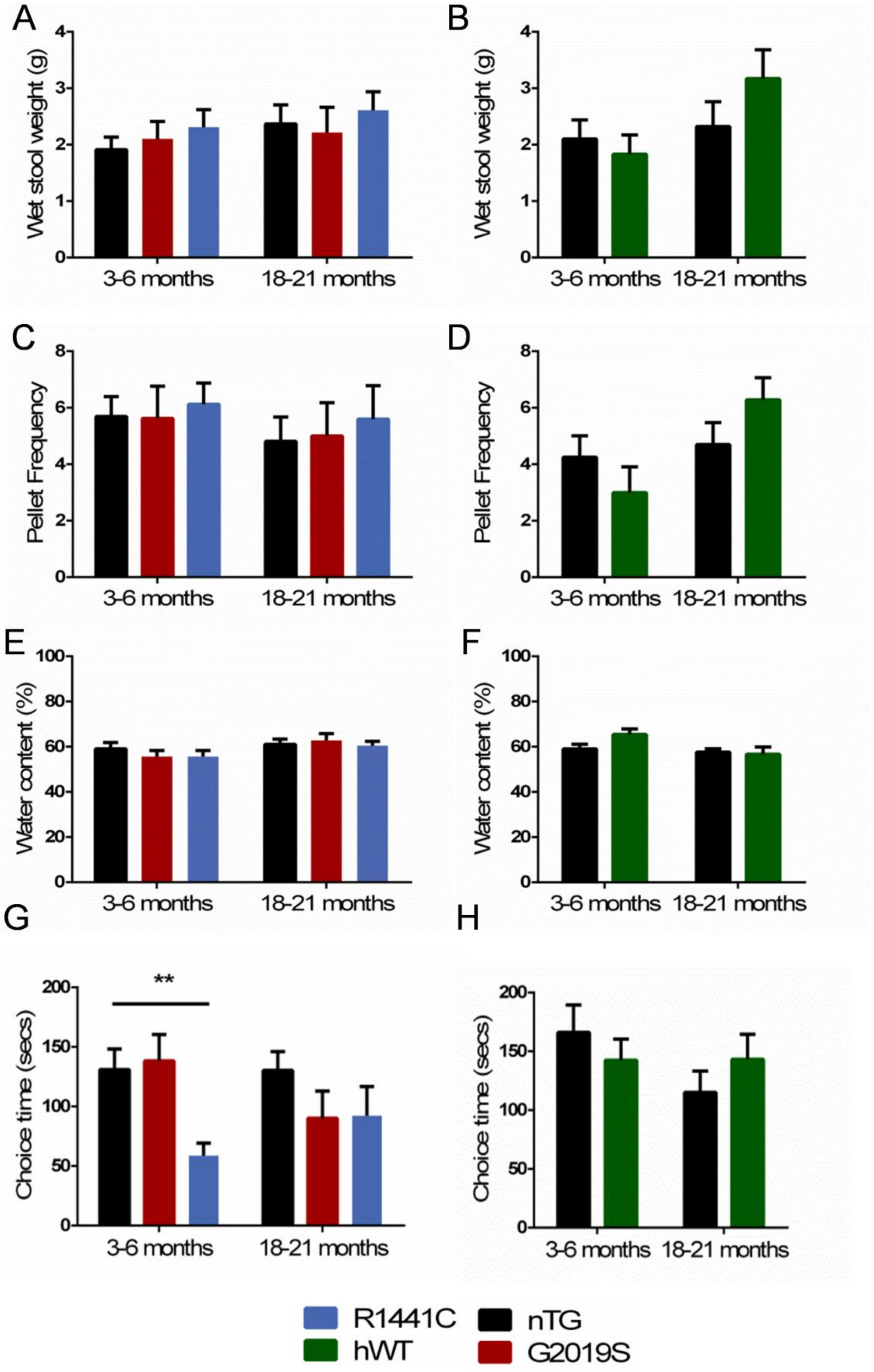
A
B
C
D
E
F
G
H
Wet stool weight (g)
Pellet Frequency
Water content (%)
Choice time (secs)
3-6 months
18-21 months
**
R1441C
hWT
nTG
G2019S

Figure S5. Mutant *LRRK2* animal stool analysis and spontaneous alternation choice times. (**A, C, E**) Neither young (one-way ANOVA: no main effect of genotype: $p > 0.05$; n=16 per genotype) nor aged (One-way ANOVA: no main effect of genotype: $p > 0.05$; n=9-14 per genotype) adult G2019S and R1441C animals showed changes in (**A**) wet stool weight, (**C**) stool pellet frequency or (**E**) percentage water content compared to age-matched nTG rats. (**B, D, E**) Neither young hWT (Welch's t-test: $p > 0.05$; n=16 per genotype) nor aged hWT (Welch's t-test: $p > 0.05$; n=9-12 per genotype) animals showed any change in (**B**) stool pellet frequency, (**D**) wet stool weight or (**F**) percentage water content compared to age-matched nTG rats**.** (**G**) Young R1441C but not G2019S animals show decreased latencies on the choice run compared to age-matched nTG rats (One-way ANOVA: main effect of genotype: $p<0.01$; n=16 per genotype). Aged G2019S and R1441C animals show no differences in run latencies compared to age-matched nTG rats (One-way ANOVA: no main effect of genotype: $p>0.05$; n=9-14 per genotype). (**H**) Neither young hWT (Welch's t-test: $p>0.05$; n=16 per genotype) nor aged hWT (Welch's t-test: $p>0.05$; n=10-11 per genotype) animals show differences in decision speed compared to age-matched nTG rats. Data are expressed as mean ± SEM. Dunnett post-hoc tests ** $p < 0.01$.

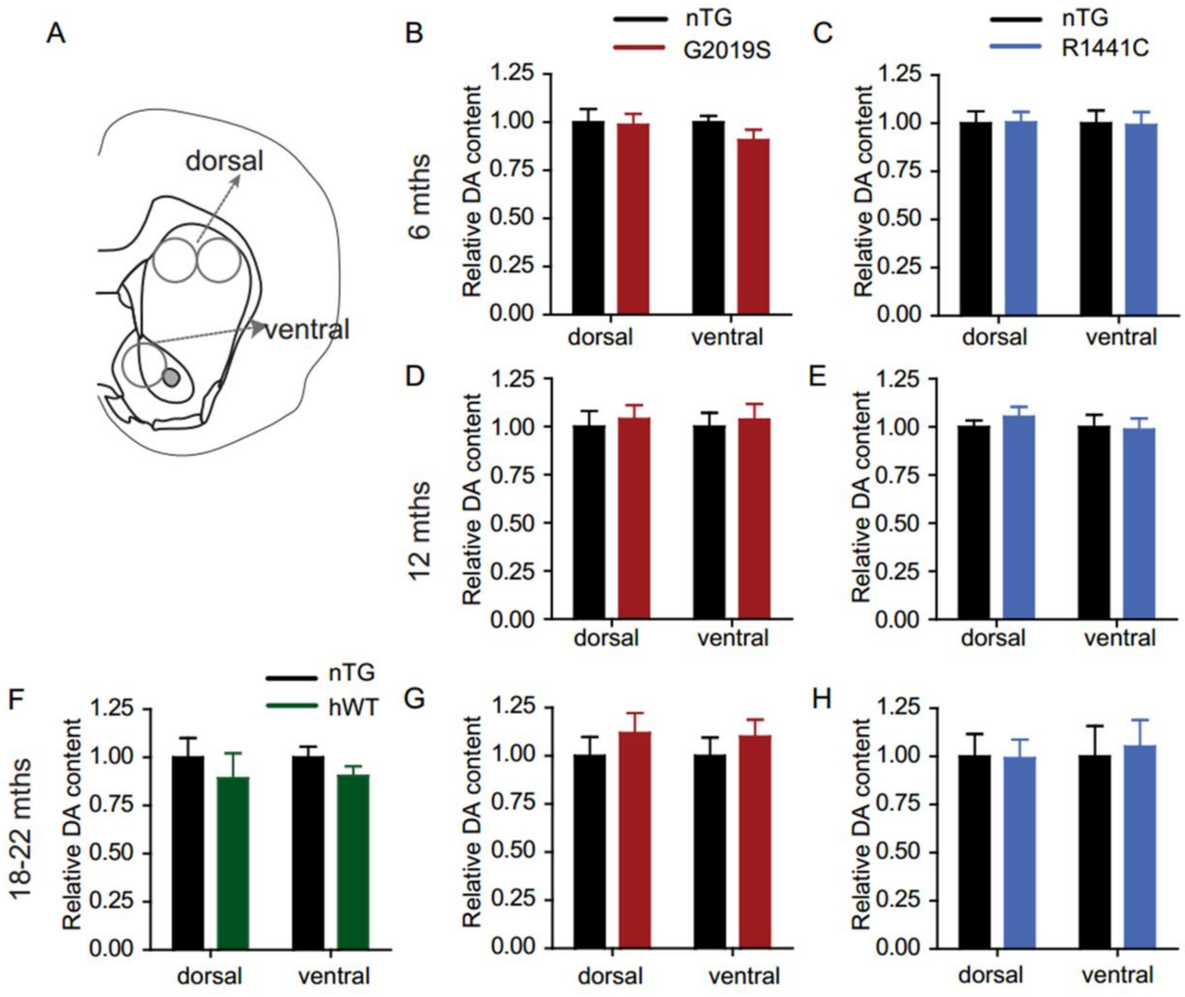

Figure S6. Striatal dopamine content is unaltered in *LRRK2* transgenic rats. (**A**) Location of tissue punches taken from striatal slices used in FCV recordings from transgenic and nTG rats. (**B-H**) Striatal dopamine content (normalized to nTG controls) in hWT, G2019S and R1441C transgenic rats and their nTG controls at ages 6, 12 and 18-22 months. No significant difference in dopamine content was found between hWT, G2019S or R1441C and nTG controls in either dorsal or ventral regions at any age ($p > 0.05$, unpaired two-tailed Mann Whitney, n= 6-10).

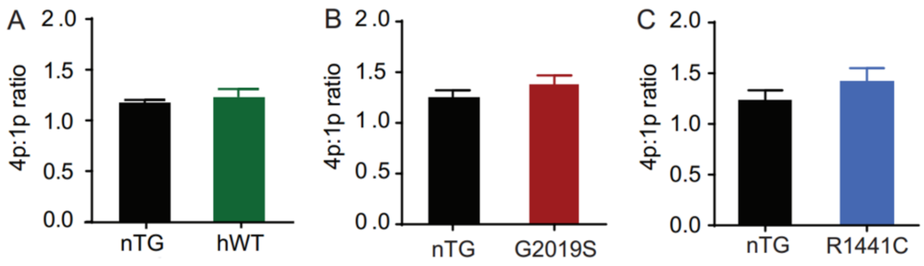

Figure S7. Short term plasticity of striatal dopamine release is unchanged in *LRRK2* transgenic rats. Ratio of $[DA]_o$ evoked by 4 pulse train stimuli at 100 Hz to that evoked by 1p (4p:1p ratio) in dorsal striatum of 18-22 month LRRK2 transgenic rats and nTG controls. No significant difference in the 4p:1p ratio was found between the nTG controls and (**A**) hWT, (**B**) G2019S or (**C**) R1441C rats ($p > 0.05$, unpaired two-tailed Mann Whitney, n=15-36).

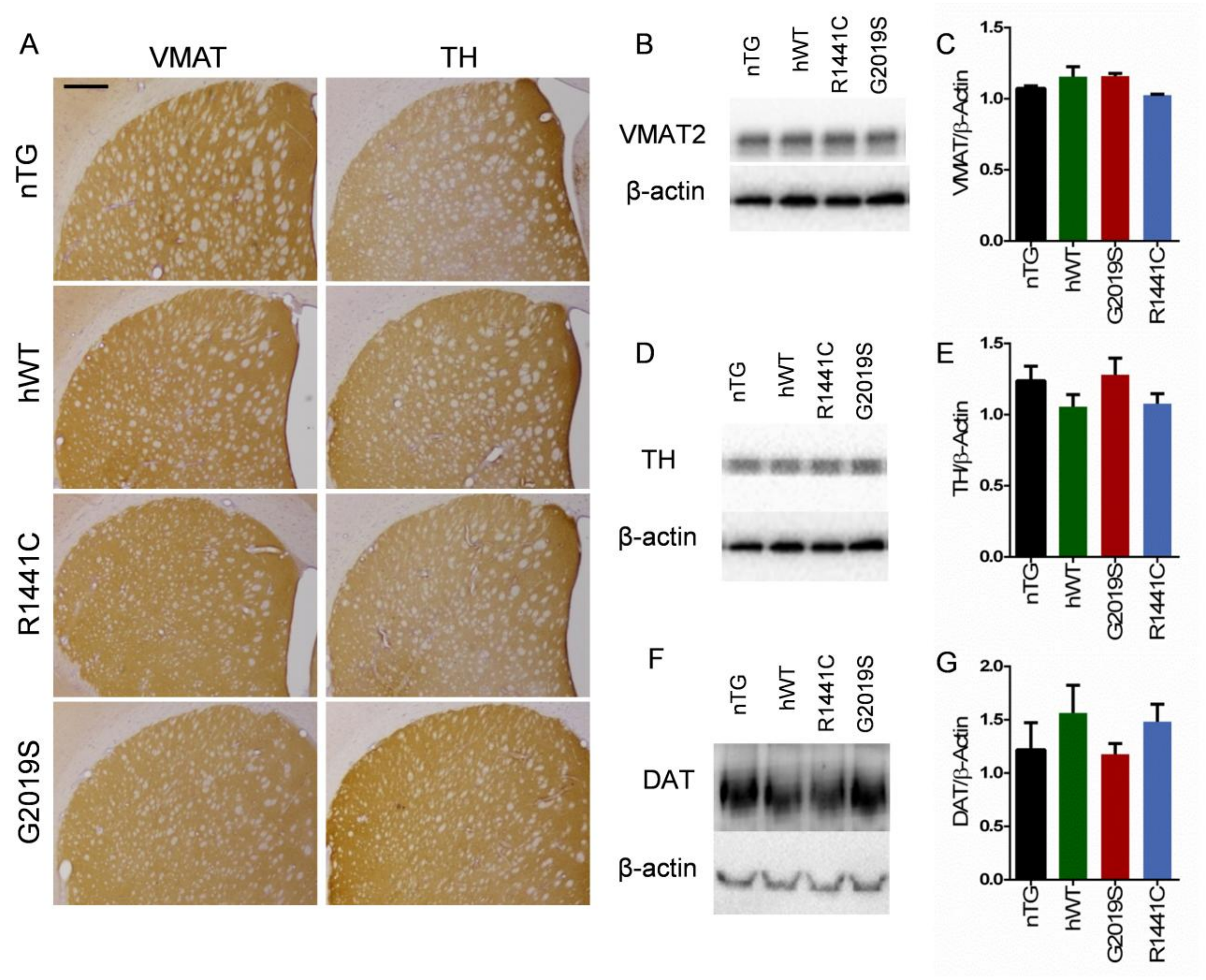

Figure S8. Dopaminergic markers in the striatum are unchanged in *LRRK2* transgenic rats. (**A**) Representative immunohistochemistry for TH and vesicular monoamine transporter in the striatum of G2019S, R1441C and hWT 18-21 month old rats showing no overt differences in expression when compared to nTG controls. Scale bar, 500 µm. (**B**) Representative Western blots for vesicular monoamine transporter in the striatum of 18-21 month old nTG, G2019S, R1441C and hWT rats. (**C**) Quantification of vesicular monoamine transporter Western blots showing no changes in the striatal vesicular monoamine transporter for G2019S, R1441C and hWT rats when compared to nTG controls (One-way ANOVA: main effect of genotype: $p > 0.05$, n=3 per genotype). (**D**) Representative Western blots for TH in the striatum of nTG,

G2019S, R1441C and hWT 18-21 month old rats. (**E**) Quantification of TH Western blots showing no changes in striatal TH for G2019S, R1441C and hWT rats at 18-21 months when compared to nTG controls (One-way ANOVA: main effect of genotype: $p > 0.05$, n=3 per genotype). (**F**) Representative Western blots for dopamine transporter in the striatum of nTG, G2019S, R1441C and hWT 18-21 month old rats. (**G**) Quantification of dopamine transporter Western blots showing no changes in striatal dopamine transporter for G2019S, R1441C and hWT rats at 18-21 months when compared to nTG controls (One-way ANOVA: main effect of genotype: $p > 0.05$, n=3 per genotype).

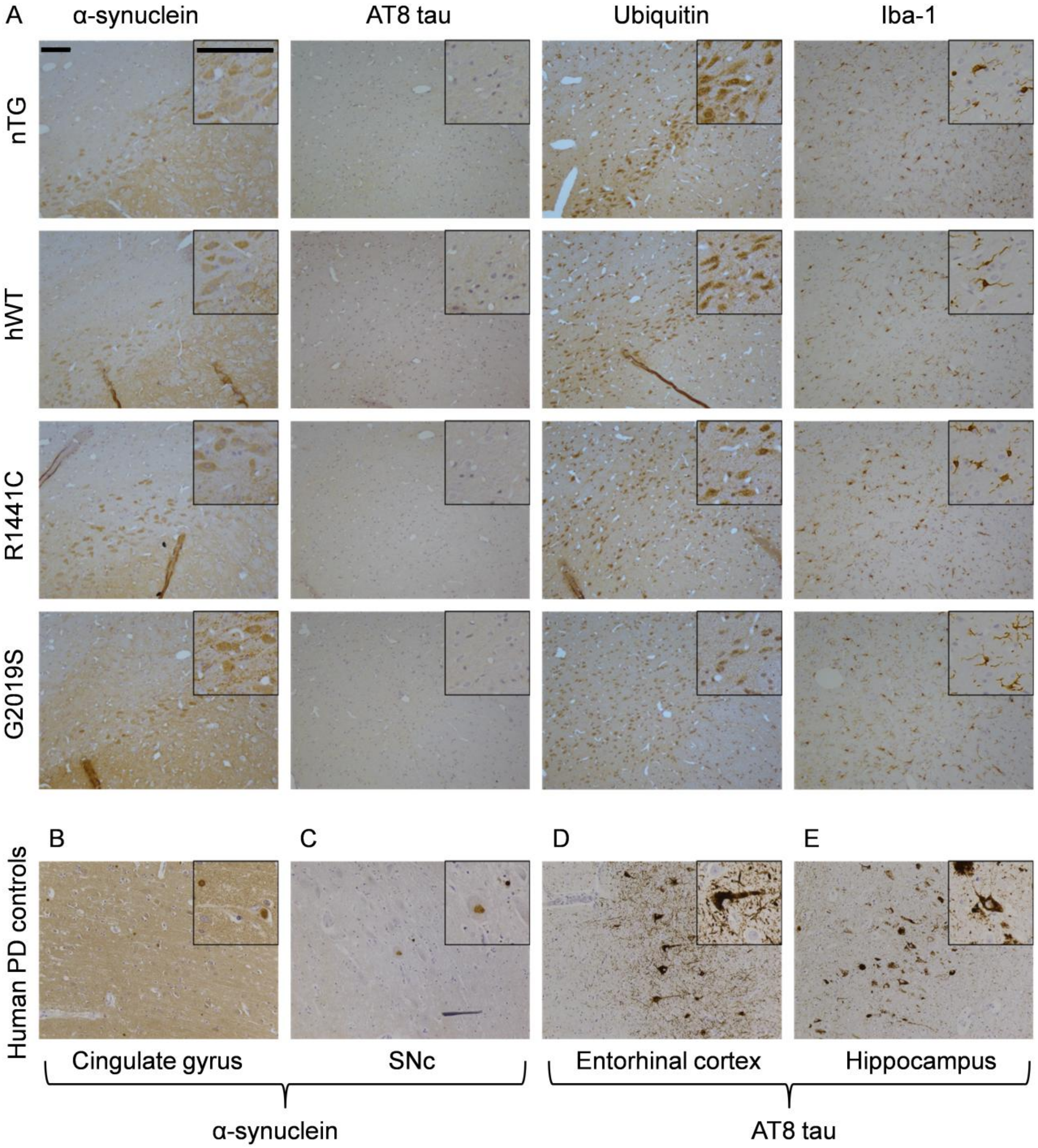

Figure S9. Immunohistochemistry for neuropathological markers in the SNc of 18-21 month old *LRRK2* rats. (**A**) Representative immunohistochemistry for α-synuclein, AT8 hyperphosphorylated tau (pser202/thr205), ubiquitin and microglial marker, Iba-1, in the SNc of nTG, G2019S, R1441C and hWT 18-21 month old rats (n≥3 per genotype). No overt

neuropathological markers were detected in mutant *LRRK2* rat brains. (**B-C**) Human post-mortem tissue from Parkinson's disease patients, positive for Lewy pathology, was stained using an α-synuclein antibody which recognizes both human and rat α-synuclein, revealing LBs in (**B**) the cingulate gyrus and (**C**) the SNc. (**D-E**) Human post-mortem tissue from Parkinson's disease patients, positive for tau tangle pathology, was stained using the AT8 antibody which recognizes both human and rat phosphorylated (ser202/thr205) tau, revealing neurofibrillary tangle pathology in (**D**) entorhinal cortex and (**E**) the hippocampus. Staining conditions used here were identical to those used in subsequent staining performed in aged rats. Scale bars, 100 μm.

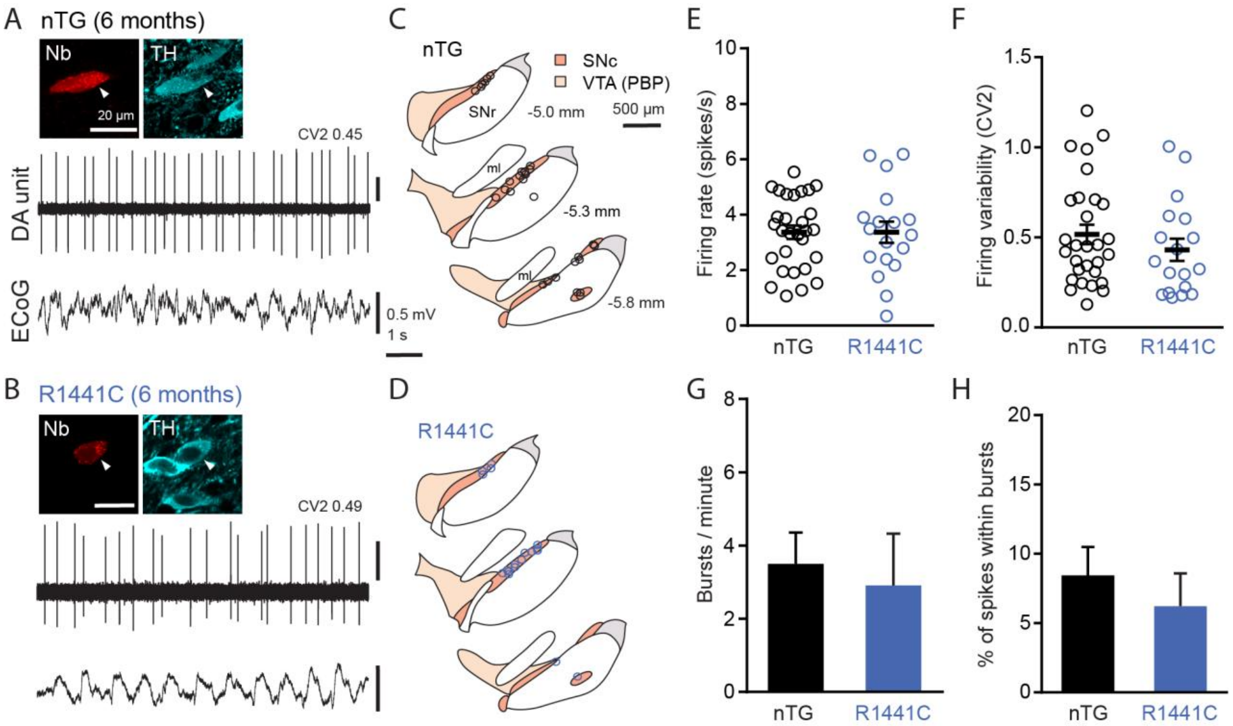

Figure S10. ***In vivo*** **firing of SNc dopamine neurons in young adult R1441C rats is similar to nTG rats.** Spontaneous activity of identified dopamine SNc neurons in 6 month old nTG (**A**) and R1441C rats (**B**) during robust slow-wave activity (measured in the electrocorticogram). Individual neurons were juxtacellularly labelled with Neurobiotin and confirmed to be dopaminergic by their expression of TH. (**C-F**) Mean firing rate (**C**), firing regularity (**D**), mean number of bursts per minute (**E**), and mean percentage of spikes occurring within bursts (**F**) were not significantly different between genotype; (nTG n=29 and R1441C n=18). Data are displayed as mean ± SEM. (**G**) Coronal schematics with approximate locations of recorded and labelled dopamine neurons for each genotype on three rostro–caudal levels (distances caudal from Bregma shown left; dorsal top, lateral right). PBP, parabrachial pigmented area of the ventral tegmental area; SNc, substantia nigra pars compacta; SNr, substantia nigra pars reticulata; ml, medial lemniscus (adapted from (48)).

Target	Product ID	Company	Concentration
DAT	AB111468	Abcam	WB (1:500)†
GFP (used to target YPet)	A11122	Life Tech.	WB (1:500)†, IHC-FF (1:250), IF (1:250)
GFP (used to target YPet)	GFP-1020	Aves Labs	IF (1:200)
Iba1	MABN92	Millipore	IHC-P (1:1000)
LRRK2	ab133474	Abcam	WB (1:500)†, IHC-FF* (1:250)
Phospho-tau (AT8) (Ser202+Thr205)	MN1020	Thermo	IHC-P (1:200)
Phospho-LRRK2 (ser910)	ab133449	Abcam	WB (1:500)†
Phospho-LRRK2 (ser935)	ab133450	Abcam	WB (1:500)†, IHC-FF* (1:200)
TH	AB152	Millipore	WB (1:1000), IHC-FF (1:2000), IF (1:500)
TH (electrophysiology)	T1299	Sigma	IHC-FF (1:1000)
Ubiquitin	Z0458	Dako	IHC-P (1:1000)
VMAT	AB1598P	Millipore	WB (1:500), IHC-FF (1:500)
α-synuclein	610786	BD Biosci.	IHC-P (1:2000)
β-Actin	ab8227	Abcam	WB (1:8000)

* Antigen Retrieval Needed † Samples not boiled

Table S1. Primary antibodies used in Western blot analysis. Abbreviations: Western blot (WB), Free floating immunohistochemistry (IHC-FF), Paraffin embedded immunohistochemistry (IHC-PF), immunofluorescence (IF).

SUPPLEMENTARY METHODS

Behavior

Inverted grid. Grip strength was assessed using the inverted grid test. Rats were placed on a 45x45 cm wire grid with wires 1.2 cm apart. The grid was held roughly 50 cm above a cushioned surface and was inverted with the rat's head facing down. The grid was then held stably until the rats released their grip. The time taken for animals to release their grip was recorded. *Stool collection.* Animals were placed in transparent cages (47x25 cm) without bedding, food or water. Fecal pellets produced over the course of 60 minutes were immediately collected in falcon tubes after expulsion. The frequency and mean weight of the pellets were determined. Pellets were then dried at 50 °C for 50 hours and reweighed to determine water content. *Catwalk.* A variety of gait parameters were assessed using the Noldus Catwalk (Noldus Information Technology). The Catwalk allows for the accurate video recording and subsequent evaluation of animals while traversing a glass panel. To ensure consistent data collection, a number of parameters were set and had to be met for a run to be accepted. Acceptable runs had to be complete within 0.5-5 s and show a maximum variance in velocity of 35%. Animals were required to perform a minimum of 5 trial runs consistent with the run criteria before data analysis was performed. Analysis was performed using Catwalk XT (v8.1; Noldus). Video scoring was performed in a semi-automated fashion. Fore- and hind-limbs were labeled via the automated tagging of video recordings and gait parameters corresponding to the mean of the trial runs were consequently calculated. Runs where limbs could not be differentiated by the software from the animal torso were discarded. *Statistical analysis.* For behavior, data were analyzed using a Welch's t-test or a parametric one- or two-way ANOVA followed by a Dunnet post hoc test and expressed as means ± SEM. Non parametric data were analyzed using a Mann Whitney U test or Kruskal–Wallis

ANOVA followed by Dunn's pairwise multiple comparison and expressed as means ± SEM. Analyses were completed using the SPSS Statistics 21 software (IBM).

Fluorescent *in situ* hybridization

Primary fibroblasts were cultured form ear clips for fluorescent in situ hybridization analysis using a protocol adapted from (49). Confluent cells were used for the generation of metaphase spreads and fluorescent in situ hybridization analysis was performed using BAC DNA as a probe.

Neuropathology

For rat tissue, animals were transcardially perfused with phosphate buffer (pH 7.4) followed by 4% paraformaldehyde. Fixed brains were paraffin-embedded using a Shandon Excelsior (Thermo), cut into 6-µm-thick sections, deparaffinised and rehydrated. Antigen retrieval was then performed using pH 6.0 citrate buffer at 100 °C (heat mediated antigen retrieval). For peroxidase-immunohistochemistry, sections were incubated overnight with primary antibodies (see Table S1) followed by a biotinylated secondary antibody which was subsequently incubated in avidin-biotin-peroxidase complex and stained using diaminobenzidine. Stained sections were counterstained using hematoxylin, dehydrated and mounted.

High-performance liquid chromatography with electrochemical detection

Dopamine content in dorsal and ventral striatum was measured by high-performance liquid chromatography with electrochemical detection as described previously (45, 46, 50, 51). Following FCV recordings, tissue punches (2 mm diameter) from the dorsal (2 punches per

hemisphere) and ventral striatum (1 punch per hemisphere) from two brain slices per animal were taken and stored at -80°C in 400 µl 0.1 M $HClO_4$. On the day of analysis, samples were thawed, homogenized and centrifuged at 13, 000 rpm for 15 min at 4°C. Samples were diluted with 0.1 M $HClO_4$ (dorsal striatum 1:10, ventral striatum 1:2). The supernatant was analyzed for dopamine content using high-performance liquid chromatography with electrochemical detection. Analytes were separated using a 4.6 x 250 mm Microsorb C18 reverse-phase column (Varian or Agilent) and detected using a Decade II SDS electrochemical detector with a Glassy carbon working electrode (Antec Leyden) set at +0.7 V with respect to a Ag/AgCl reference electrode. The mobile phase consisted of 13% methanol (v/v), 0.12 M NaH_2PO_4, 0.5 mM OSA, 0.8 mM EDTA, at pH 4.6, and the flow rate was fixed at 1 ml/min. Analyte measurements were normalized to tissue punch volume.
